# Integrating temporal and spatial control of electronic transitions for bright multiphoton upconversion

**DOI:** 10.1038/s41467-019-09850-2

**Published:** 2019-04-18

**Authors:** Tianying Sun, Yuhua Li, Wai Lok Ho, Qi Zhu, Xian Chen, Limin Jin, Haomiao Zhu, Bolong Huang, Jun Lin, Brent E. Little, Sai Tak Chu, Feng Wang

**Affiliations:** 1 Department of Materials Science and Engineering, City University of Hong Kong, 83 Tat Chee Avenue, Hong Kong, SAR China; 2 City University of Hong Kong Shenzhen Research Institute, Shenzhen, 518057 China; 3 Department of Physics, City University of Hong Kong, 83 Tat Chee Avenue, Hong Kong, SAR China; 4 State Key Laboratory on Tunable laser Technology, Ministry of Industry and Information Technology Key Lab of Micro-Nano Optoelectronic Information System, Shenzhen Graduate School, Harbin Institute of Technology, Shenzhen, 518055 China; 5 Xiamen Institute of Rare-earth Materials, Haixi Institutes, Chinese Academy of Sciences, Xiamen, Fujian, 361000 China; 6 Department of Applied Biology and Chemical Technology, The Hong Kong Polytechnic University, Hung Hom, Hong Kong, SAR China; 7 State Key Laboratory of Rare Earth Resource Utilization, Changchun Institute of Applied Chemistry, Chinese Academy of Sciences, Changchun, 130022 China; 8 State Key Laboratory of Transient Optics and Photonics, Xi’an Institute of Optics and Precision Mechanics, Chinese Academy of Sciences, Xi’an, 710119 China

**Keywords:** Nanoparticles, Integrated optics, Lithography

## Abstract

The applications of lanthanide-doped upconversion nanomaterials are limited by unsatisfactory brightness currently. Herein, a general strategy is proposed for boosting the upconversion efficiency in Er^3+^ ions, based on combined use of a core−shell nanostructured host and an integrated optical waveguide circuit excitation platform. A NaErF_4_@NaYF_4_ core−shell nanoparticle is constructed to host the upconversion process for minimizing non-radiative dissipation of excitation energy by surface quenchers. Furthermore, an integrated optical microring resonator is designed to promote absorption of excitation light by the nanoparticles, which alleviates quenching of excited states due to cross-relaxation and phonon-assisted energy transfer. As a result, multiphoton upconversion emission with a large anti-Stokes shift (greater than 1150 nm) and a high energy conversion efficiency (over 5.0%) is achieved under excitation at 1550 nm. These advances in controlling photon upconversion offer exciting opportunities for important photonics applications.

## Abstract (web summary)

Here, the authors control concentration quenching in a stoichiometric Er3+ compound using a core−shell nanostructured host and an integrated optical waveguide circuit excitation platform. The efficient telecom to ultraviolet upconversion allows for exploration of photonic applications involving upconversion nanomaterials.

## Introduction

**Fig. 1:**
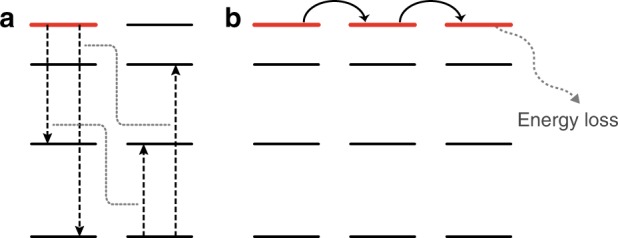
General processes of dopant interactions responsible for depopulation of an excited state. **a** Cross-relaxation and phonon-assisted energy transfer that depopulates an excited state locally. To counteract the depopulation associated with the localized energy exchange interaction, a high excitation power is needed to enhance the excitation process. **b** Long-distance energy migration through the dopant sublattice that takes the energy to lattice or surface defects. The energy migration-induced depopulation can be alleviated by spatially confining the excitation energy

Lanthanide-doped upconversion nanoparticles that convert low energy excitation into higher-energy emissions are important for applications in diverse fields ranging from life science to information technology. By integrating upconversion nanoparticles into various systems, localized visible and ultraviolet (UV) emissions can be generated upon near-infrared (NIR) excitation. The effect has enabled precise and remote control of biochemical reactions as well as the creation of full-color 3D displays^1–7^. Nevertheless, despite of the unique optical properties, wider application of upconversion processes has been hindered by limited emission intensity due to low concentration of optical centers (or dopants) in most nanoparticles. When dopant concentration increases, interactions of optical centers become significant as a result of reduced inter-dopant distance, causing depopulation of excited states and thus attenuation in the upconversion emission (Fig. 1).

Attempts have been made to alleviate concentration quenching so that unusually high dopant concentrations can be used to boost upconversion emission intensity. By promoting the excitation process through the use of high excitation irradiance (~10^6^ W cm^−2^) and dye sensitization, depopulations of excited states are compensated and lead to elevated quenching concentrations of Tm^3+^, Er^3+^, and Nd^3+^ activators^8–10^. Core−shell nanostructural engineering has also been exploited to mitigating concentration quenching by eliminating dissipation of energy to lattice and surface defects. Using this method, concentration quenching in both sensitizer (Yb^3+^) and activator (Er^3+^) ions is greatly suppressed^11–17^. Despite these research efforts, photon upconversion at a high concentration of activators is usually dominated by low-order processes featuring emission in the spectral region of long wavelengths. Challenges remain for achieving highly efficient multiphoton upconversion with emissions in the short-wavelength regime by using low power excitation. Particularly, there are no known effective approaches to achieve efficient UV emission under excitation in the wavelength range for optical communication, where many inexpensive and high-performance lasers and optical components can be readily acquired from the mature telecommunications industry.

Here, we present rational control of concentration quenching in a stoichiometric Er^3+^ compound by combined use of a core−shell nanostructured host and an integrated optical waveguide circuit excitation platform. We show an experimental validation of enhancing multiphoton UV upconversion under excitation at 1550 nm, corresponding to an anti-Stokes shift of over 1150 nm, through collective control of intra-particle energy transfer and excitation method. By taking advantage of the efficient 1550 nm-to-UV upconversion, we further demonstrate a novel technique for precise formation of polymer waveguides and periodic patterns with the upconverted UV emission.

## Results

### Synthesis and characterization

**Fig. 2:**
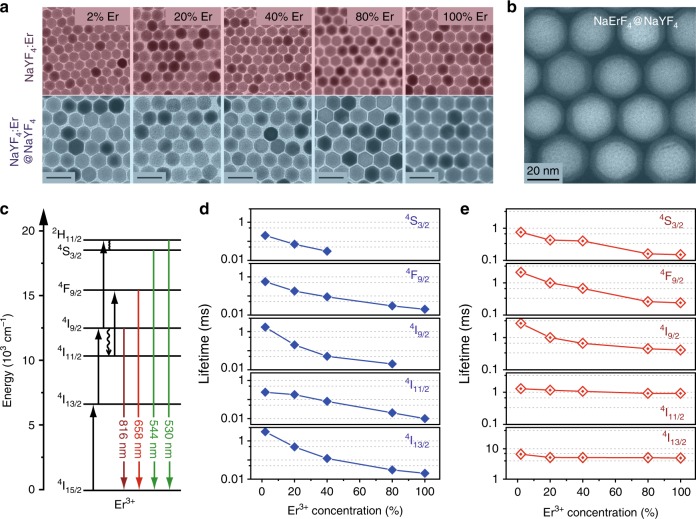
Comparative characterization of the NaYF_4_:Er and NaYF_4_:Er@NaYF_4_ nanocrystals. **a** TEM images of the NaYF_4_:Er (2−100%) core and the NaYF_4_:Er (2−100%)@NaYF_4_ core−shell nanocrystals. Scale bars are 50 nm. **b** HAADF scanning TEM image of the NaErF_4_@NaYF_4_ nanoparticle highlighting the core−shell structure. **c** Proposed energy diagram showing upconversion processes in Er^3+^ under 1532 nm excitation. The full, wavy, and colored arrows represent excitation, multiphonon relaxation, and emission processes, respectively. **d**, **e** Lifetimes of various excited states of Er^3+^ as a function of the dopant concentration in the NaYF_4_:Er (2−100%) core and the NaYF_4_:Er (2−100%)@NaYF_4_ core−shell nanoparticles, respectively. The excitation pulse energy density was set at 1 mJ mm^−2^ for decay measurements

We first investigated the behaviors of concentration quenching in different energy states of Er^3+^, which has not been clearly delineated^18^. To this end, we conduct a comparative investigation of a series of NaYF_4_:Er (2**−**100%) nanoparticles with and without a protection layer of NaYF_4_. The inert NaYF_4_ shell is able to selectively suppress energy loss to lattice defects and surface quenching sites that arises from energy migration through the dopant sublattice^19^. Assessment of the core−shell nanoparticles thereby allows us to probe the quenching mechanism. The nanoparticles were synthesized by a layer-by-layer epitaxial growth protocol (Supplementary Fig. 1)^20^. Figure 2a shows transmission electron microscopy (TEM) images of the nanoparticles comprising different concentration of Er^3+^, revealing highly uniform size and shape across the whole concentration series. High-angle annular dark-field (HAADF) scanning TEM image of a representative sample shows distinguished Z-contrast between the NaErF_4_ and NaYF_4_ layers (Fig. 2b), clearly revealing the core−shell nature of the nanoparticles. The X-ray powder diffraction (XRD) and high-resolution TEM (Supplementary Figs. 1 and 2) measurements further confirm high crystallinity of the nanoparticles with a single hexagonal phase. The consistent structural feature qualifies these nanoparticles as model platforms for investigating the effects of dopant concentration on upconversion properties.

### Concentration quenching effect

We studied the lifetimes of several energy states of Er^3+^, which are all involved in the upconversion process and may be subject to concentration quenching (Fig. 2c and Supplementary Fig. 3). Because the decay curves were found to display an appreciable dependence on excitation power density due to intra-particle energy transfer (Supplementary Fig. 4), the dopant concentration-induced changes in lifetimes were examined under the same excitation power density. Figure 2d shows the lifetimes for the major excited states of Er^3+^ as a function of dopant concentration in the core nanoparticles (see Supplementary Fig. 5 for the decay curves). In accord with concentration-induced quenching, the lifetimes for the excited states under study all drop quickly with increasing Er^3+^ concentration, which is consistent with the steady-state spectral measurement of the core nanoparticles. By contrast, the NaYF_4_-coated core−shell counterparts showed much longer lifetimes especially when Er^3+^ concentrations are high (Fig. 2e), indicating largely alleviated quenching processes.

**Fig. 3:**
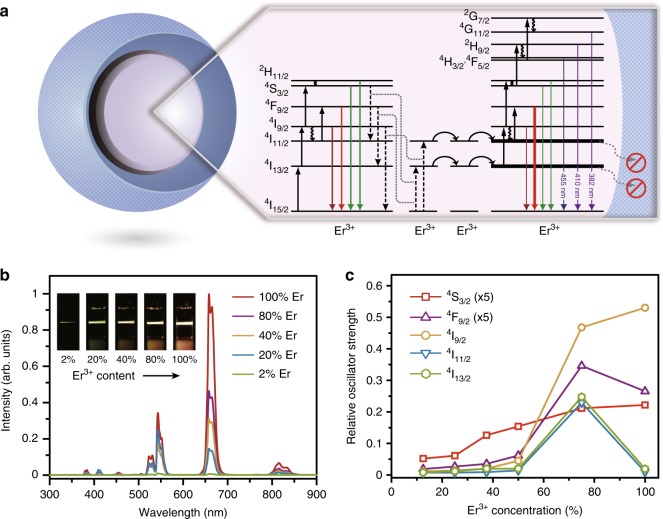
Concentration quenching in the NaYF_4_:Er@NaYF_4_ nanocrystals. **a** Proposed concentration quenching processes in NaErF_4_@NaYF_4_ core−shell nanoparticles by 1532 nm excitation. The full, wavy, and colored arrows represent excitation, multiphonon relaxation, and emission processes, respectively. **b** Emission spectra of NaYF_4_:Er (2−100 %)@NaYF_4_ nanoparticles in cyclohexane dispersions (0.01 M) as a function of Er^3+^ concentration. All spectra were recorded under excitation of a 1532 nm CW diode laser at a power density of 21 W cm^−2^. Inset: luminescence photographs of the corresponding nanoparticle colloids. **c** The relative oscillator strengths for different excited states as a function of Er^3+^ concentration. Note that the concentrations in the theoretical calculation may not be exactly the same as that used in the experiments but still correctly illustrate the evolution of electronic transition probabilities

It is noted that the dependence of lifetime on Er^3+^ concentration is not uniform for different excited states (Fig. 2e). Therefore, concentration quenching of individual excited state is dominated by different processes (Fig. 3a). Lifetimes of the ^4^I_11/2_ and ^4^I_13/2_ states are mostly preserved as Er^3+^ concentration increases, revealing a major role of energy migration to quenching centers in depopulating these two states. Core−shell nanostructures confine energy migration and prevent energy trapping by quenching centers^21,22^. In comparison, decay rates of the ^4^S_3/2_, ^4^F_9/2_, and ^4^I_9/2_ states are appreciably accelerated by elevating Er^3+^ concentrations. The lifetime gradient was unlikely to level off by enhancing surface protection through the use of thicker shells (Supplementary Figs. 6−8). Thus, these three states largely suffered from depopulation by cross-relaxation and phonon-assisted energy transfer, which are localized processes and remain active in nanostructured hosts.

The steady-state spectra agree with the time decay studies that not all the excited states were equally protected in the core−shell nanoparticles (Fig. 3b and Supplementary Fig. 8b). In line with effective suppression of depopulation in the ^4^I_11/2_ and ^4^I_13/2_ states, the emission originating from the ^4^F_9/2_ state (658 nm) was considerably enhanced at high Er^3+^ concentrations. By contrast, emissions at short wavelengths are only marginally intensified, resulting in decreased relative intensity ratio of green to red emissions at elevated Er^3+^ concentrations. The observations are ascribed to quenching of the ^4^S_3/2_, ^4^F_9/2_, and ^4^I_9/2_ states by cross-relaxation and phonon-assisted energy transfer (Fig. 3a). As these intermediate states are critical for establishing the population in the higher-lying ^2^H_9/2_ and ^4^G_11/2_ states, the blue and violet emissions are extremely weak relative to the red emission. Concentration quenching of the emitting states (^2^H_9/2_ and ^4^G_11/2_) also accounts for the weak emissions at the short-wavelength end (Supplementary Fig. 9).

To shed more light on the concentration quenching effect, we calculated the relative oscillator strength (ROS) of each excited state as a function of Er^3+^ concentration on the overall core−shell model^23^. In general, the ROS grows near-linearly as Er^3+^ concentration increases to 50% (Fig. 3c), which is ascribed to increasing number of optical centers in the nanoparticles. With further increase of Er^3+^ concentration to 75%, abnormal increases of the ROSs were noted for the ^4^F_9/2_, ^4^I_9/2_, ^4^I_11/2_, and ^4^I_13/2_ states. The leap of the ROS corroborates de-excitation of the higher-lying excited states due to concentration quenching, which accounts for the elevated population in the low-lying ones. The drop of ROSs for the ^4^I_11/2_ and ^4^I_13/2_ states at a substantially high Er^3+^ concentration (100%) is attributed to the enhancement of energy transfer upconversion that depletes these two states (Supplementary Fig. 10)^13^.

The mechanistic investigation reveals that the use of core−shell nanostructure alone is unable to alleviate concentration quenching in all the energy states of Er^3+^. In order to achieve efficient multiphoton upconversion luminescence, joint use of high excitation power is also crucial to counteract the depopulation of the high-lying intermediate states. In an upconversion process, an intermediate state is depopulated by being excited to a higher-lying state in addition to linear decay to lower-lying states. The excitation process is pump power dependent and thus upconversion emission dominates over luminescence quenching under high excitation powers^24^.

### Microring resonator-assisted excitation of upconversion

**Fig. 4:**
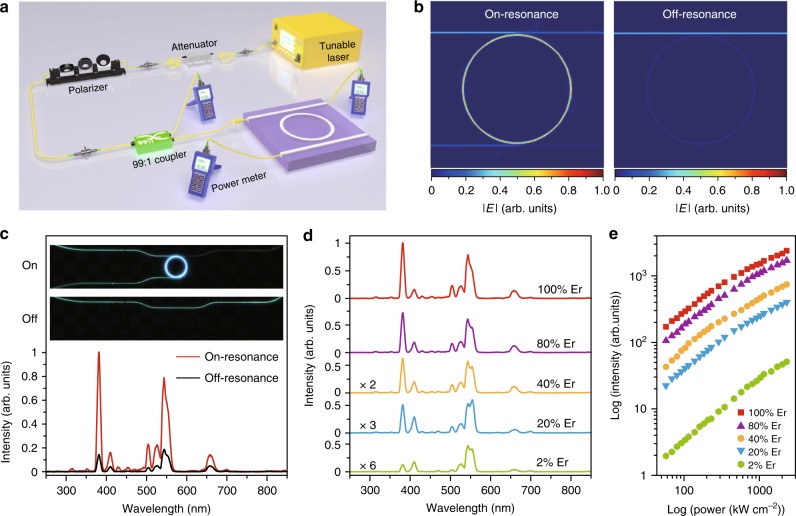
Microring resonator-assisted excitation of NaErF_4_@NaYF_4_ nanocrystals. **a** Schematic diagram of the set-up for the microring resonator-assisted excitation scheme. **b** Electrical field distribution in the waveguide structure when the incident light is in resonance (left) and out of resonance (right) with the microring resonator. **c** Emission spectra and optical micrographs (inset) recorded from an identical specimen in different resonance states. **d** Emission spectra of NaYF_4_:Er (2−100%)@NaYF_4_ nanoparticles as a function of Er^3+^ concentration under microring resonator-assisted excitation (1549.47 nm, 2300 kW cm^−2^). **e** Upconversion emission intensity at 382 nm as a function of excitation power density for NaYF_4_:Er (2−100%)@NaYF_4_ on microring resonator, demonstrating attenuation of UV emission with decreasing Er^3+^ contents

In view of the small absorption cross-section of the lanthanide ions (~10^−21^ cm^2^)^25^, conventional approaches for enhancing the upconversion rates are focused on increasing the excitation intensity and/or localizing the illumination field. Attempts have been made to modulate excitation field around upconversion nanoparticles by surface-plasmon coupling and photonic-crystal engineering^26,27^. However, these methods need tedious processing of upconversion nanoparticles and the amplification of optical field may prove to be limited. Here we propose a more effective approach to enhance the upconversion by using an optical microring resonator excitation platform (Fig. 4a). The integrated waveguide circuit increases the interaction length between the excitation photons with the nanoparticles. When it is on resonance, the excitation light circulates in the microring resonator by a number of times proportional to its *Q*-factor. As the circulating light cumulates, the optical field in the microring resonator can also be enhanced by orders of magnitude^28^. Thus, the chance for the photon to be absorbed by the nanoparticles will be markedly increased.

The microring resonator used in this work is composed of high-index doped silica glass that is semi-buried within a SiO_2_^29^. In this application, the surface of the chip is polished flat with the surface of the core waveguide exposed (Supplementary Fig. 11). Upconversion nanoparticles are applied on the surface of the substrate and excited by evanescent fields of the waveguides. A narrow linewidth tunable continuous-wave laser is used to tune the input excitation on/off resonance of the resonator. While on resonance, the input signal circulates within the resonator in phase, thereby affording a much stronger electric field relative to that in the straight waveguide (Fig. 4b). Importantly, due to high wavelength-selectivity of the ring resonator (Supplementary Fig. 12), circulation of incident light can be disabled by subtle detuning of wavelength (<0.01 nm), which permits in situ examination of the enhancement effect^28–35^.

Figure 4c depicts upconversion property of NaErF_4_@NaYF_4_ core−shell nanoparticles sitting on the waveguide structure. At a constant input power of 20 mW, the microring resonator provided a largely amplified excitation density with respect to the straight bus waveguide (2300 versus 133 kW cm^−2^) (Supplementary Fig. 13). Accordingly, upconversion luminescence, especially emission in the short-wavelength region, was appreciably enhanced when the resonance status was changed by tuning the wavelength of incident light from 1550.00 nm (off-resonance) to 1549.47 nm (on-resonance). Correspondingly, we recorded an increase of energy conversion efficiency from 1.1 to 5.0% (Supplementary Fig. 14). The results clearly support reduced quenching in the intermediate states at a high excitation power density. It is worth noting that the actual energy conversion efficiency offered by the ring resonator is underestimated by the current experimental setup, which inevitably suffers from interference of the less efficient upconversion processes occurring at the long bus waveguide (inset of Fig. 4c). Due to effective alleviation of concentration quenching, UV lasing was achieved from NaErF_4_@NaYF_4_ nanoparticles incorporated into a microdisk laser cavity (Supplementary Fig. 15)^36^.

The microring resonator circuit is versatile for providing amplified excitation to upconversion particles, without the need for controlling their assembly on the waveguide (Supplementary Fig. 16). However, the magnitude of upconversion enhancement was found to depend on nanoparticle composition and input power (Supplementary Fig. 17). In general, the enhancement effect weakens with increasing excitation power and Er^3+^ concentration due to saturation of upconversion emissions in the high-power regime (Supplementary Fig. 17b). However, the use of highly Er^3+^-doped nanoparticles is essential for achieving bright multiphoton upconversion in the short-wavelength regime. As Er^3+^ concentration decreases, both UV emission intensity and UV-to-visible intensity ratio decrease rapidly at the same excitation power (Fig. 4d, e). The observations are ascribed to a reduction in density of optical carriers, which diminish the absorption of surplus excitation light. A low Er^3+^ content also disfavors interionic interaction and thus inhibits the efficient energy transfer upconversion process.

### Micropatterning through upconversion

**Fig. 5:**
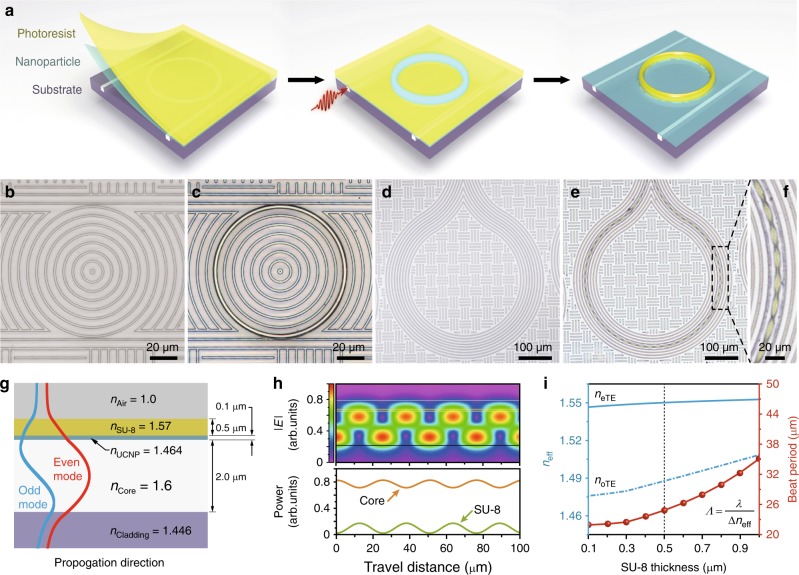
Photolithography with the UV emission for precise formation of waveguides and patterns. **a** Schematic design for patterning SU-8 photoresist. NaErF_4_@NaYF_4_ upconversion nanoparticles and SU-8 film are deposited on the substrate. Locally generated upconversion light by the waveguide structure induces selective exposure of SU-8 film. **b**, **c** Micrographs of a microring resonator before and after developing a SU-8 layer on the ring. **d**–**f** Micrographs of a waveguide loop before and after developing a periodic structure of SU-8. **g** Waveguide geometry and parameters of the simulation model with the upconversion nanoparticle (UCNP) and SU-8 upper cladding layers. **h** Distribution of the electric field amplitude along the propagation direction showing the beating between the odd and even modes (top) and the plot of fractional power in the core and SU-8 regions of the waveguide as a function of propagation distance (bottom). **i** Calculated effective indices of the even/odd modes and the beat period as a function of SU-8 thickness at 1550 nm, respectively. The period observed in (**f**) matches well with the calculated period *Λ* = *λ*/*Δn*_eff_

The availability of intense UV emission from the surface of the waveguide structures offers a great opportunity for microfabrication of fine structures and patterns on them to modify their optical responses or to form advanced devices when incorporated with functional polymers^37–44^. As a proof of concept, we demonstrate the precise fabrication of an SU-8 polymer waveguide on top of the microring resonator (Fig. 5a). The procedure involves sequential deposition of upconversion nanoparticles and SU-8 on the resonator substrate by drop-casting and spin-coating, respectively (Supplementary Fig. 18). Selective exposure of the SU-8 film is achieved by the locally upconverted UV light in the region of the ring resonator under on-resonance excitation (Fig. 5b). Our study shows that curing of a 2 μm thick SU-8 waveguide can be accomplished in <10 min by excitation of only 20 μW near 1550 nm. After developing, the SU-8 copies the ring structure well on the substrate with a perfect alignment and a smooth surface (Fig. 5d and Supplementary Fig. 19). Notably, this approach is simple and fast for controllable curing of photoresists without the need for a photomask or control of laser scanning, which provides a powerful addition to existing techniques for photopatterning of polymers^45,46^.

The same methodology can be adopted to fabricate periodic structures on waveguides utilizing their own interference patterns. By using a simple channel waveguide with core index (*n* = 1.60) that is slightly higher than the SU-8 index (*n* = 1.57) (Supplementary Fig. 20), strong intensity patterns from the coupling between its odd and even modes were created and modulated the solidification of the SU-8 (Fig. 5d–f). The observed period at an SU-8 thickness of 0.5 μm is around 25 μm (Fig. 5f), which is in consistence with the calculated beat period (*Λ*) by the FEM method (Fig. 5g–i). In agreement with the beating between the odd and even modes of the waveguide, we further achieved tuning of the pattern period by controlling the thickness of the SU-8 coating (Supplementary Fig. 21). These findings demonstrate one promising approach for rapid fabrication of periodic structures through photolithography.

## Discussion

The investigation of photon upconversion in a core−shell nanostructured NaErF_4_ crystal under an integrated optical waveguide circuit excitation platform enables improved understanding and control of concentration quenching. The ability to mitigate complex concentration quenching in a multitude of lanthanide excited states consolidates the position of using high dopant concentration as a versatile approach for enhancing multiphoton upconversion. On a separate note, the realization of efficient UV emission by excitation in the wavelength range for optical communications would largely promote photonic applications of upconversion nanomaterials.

## Methods

### Nanoparticle synthesis

We synthesized the NaYF_4_:Er@NaYF_4_ nanoparticles using the method of ref. ^20^. Additional experimental details are provided in the Supplementary Note 1.

### Theoretical modelling

The oscillator strengths were derived from calculated electric-dipole transitions by time-dependent density functional theory (TD-DFT). To perform the excited state calculations, we chose the two-electron based Tamm-Dancoff approximation imported from self-consistently corrected ground state wavefunctions^23^. β-NaREF_4_ comprising different amount of Er^3+^ dopants (12.5–100%) were examined by a series of TD-DFT calculations. The electrical field in the microring resonator was simulated by three-dimensional finite-difference time domain (3D-FDTD) method. Finite element method (FEM) was used to calculate the modal propagation constants and field profiles of E-field.

### Physical measurement

TEM and HAADF scanning TEM were carried out on a JEM-2100F transmission electron microscope at an acceleration voltage of 200 kV. The upconversion emission spectra were recorded with a Hitachi F-4600 spectrophotometer. The decay curves were measured by an Edinburgh FLSP920 spectrometer. Optical micrographs were recorded with an advanced research microscope (ECLIPSE Ni-U, Nikon). All measurements were performed at room temperature.

## Supplementary information

Supplementary Information

## Data Availability

The data that support the findings of this study are available from the corresponding author upon request.
