# Supplementary material for: Integrating temporal and spatial control of electronic transitions for bright multiphoton upconversion
Source: Nat Commun. 2019 Apr 18;10:1811. doi: 10.1038/s41467-019-09850-2 (PMC6472381; doi:10.1038/s41467-019-09850-2)
Supplement: Supplementary file 1 — Supplementary Information [file 41467_2019_9850_MOESM1_ESM.pdf]

Supplementary Information

Integrating Temporal and Spatial Control of Electronic Transitions for Bright Multiphoton Upconversion

Wang et al.

Supplementary Note 1: Experimental Details

Reagents: $Y(CH_3CO_2)_3 \cdot xH_2O$ (99.9%), $Er(CH_3CO_2)_3 \cdot xH_2O$ (99.9%), $Lu(CH_3CO_2)_3 \cdot xH_2O$ (99.9%), NaOH (>98%), NH_4F (>98%), trisodium citrate, 1-octadecene (ODE) (90%), oleic acid (OA) (90%), were all purchased from Sigma-Aldrich. Absolute ethanol (99.85%), methyl alcohol (99.99%), and cyclohexane (99.9%) were purchased from VWR International. SU-8 2002, SU-8 2000.5 and SU-8 developer were purchased from Microchem. All chemicals were used as received without further purification.

Synthesis of $NaYF_4$:Er core nanoparticles. The $NaYF_4$:Er core nanoparticle was synthesized by using our previously established protocol.[1] Firstly, 4 mL $RE(CH_3CO_2)_3$ (0.2 M, RE = Y and Er) in water solution was added to a binary solvent mixture of OA (6 mL) and ODE (14 mL) in a 50 mL flask. The mixture was heated at 150 ^{o}C for 50 min before cooling down to 45 ^{o}C. Shortly thereafter, an appropriate amount (7.2, 7.1, 7.0, 6.9, and 6.8 mL for 2%, 20%, 40%, 80%, and 100% Er, respectively) of NH_4F (0.4 M) and 2 mL of NaOH (1 M) in methanol solution was added, and the resultant solution was stirred for 90 min. After the methanol was evaporated, the solution was heated to 290 ^{o}C under argon protection for 1 h and then cooled down to room temperature. The resulting nanoparticles in mixture were precipitated by addition of ethanol, collected by centrifugation at 6000 rpm for 3 min, washed with ethanol and methanol for several times, and finally re-dispersed in 4 mL cyclohexane for further use.

Synthesis of $NaYF_4$:Er@$NaYF_4$ core–shell nanoparticles. The $NaYF_4$:Er@$NaYF_4$ core–shell nanoparticles with a mean shell thickness of 3 nm were synthesized by an epitaxial growth protocol that uses preformed core nanoparticles as seeds to mediate the shell growth.[1] $NaYF_4$ shell precursor was first prepared by mixing an aqueous solution of $Y(CH_3CO_2)_3$ (4 mL, 0.2 M) with OA (6 mL) and ODE (14 mL) in a 50 mL flask. The mixture was heated at 150 ^{o}C for 50 min before cooling down to 45 ^{o}C. Subsequently, pre-synthesized $NaYF_4$:Er core nanoparticles along with an appropriate amount (same as those used in the core nanoparticle synthesis) of NH_4F (0.4 M) and 2 mL of NaOH (1 M) in methanol solution was added, and the resultant solution was stirred for 90 min. After the methanol was evaporated, the solution was heated to 290°C under argon protection for 1 h and then cooled down to room temperature. The resulting nanoparticles in the mixture were precipitated by addition of ethanol, collected by centrifugation at 6000 rpm for 3 min, washed with ethanol and methanol for several times, and finally re-dispersed in 4 mL cyclohexane.

Synthesis of thick shelled (7 and 14 nm) $NaYF_4$:Er@$NaYF_4$ nanoparticles. The $NaYF_4$:Er@$NaYF_4$ core–shell nanoparticles with thick $NaYF_4$ shells were synthesized by a successive hot-injection method. $NaYF_4$ shell precursors were first prepared by mixing an aqueous solution of $Y(CH_3CO_2)_3$ (20 mL, 0.2 M) with OA (30 mL) and ODE (70 mL) in a 250 mL flask. The mixture was heated at 150 ^oC for 50 min before cooling down to 45 ^oC. Subsequently, 40 mL NH_4F (0.4 M) and 10 mL of NaOH (1 M) in methanol solution was added, and the resultant solution was stirred overnight to allow the full evaporation of methanol. To prepare the $NaYF_4$:Er@$NaYF_4$ core–shell nanoparticles with a mean shell thickness of 7 nm, as-prepared $NaYF_4$:Er@$NaYF_4$ (3 nm) core–shell nanoparticles (0.4 mL) in cyclohexane dispersion were added into a 50 mL flask together with 6 mL OA and 14 mL ODE. The mixture was heated at 290 ^oC under argon atmosphere. $NaYF_4$ shell precursors were then quickly injected using a syringe pump at every 10 min (0.25 mL each time). The injection was repeated for 20 times and the temperature was kept for another 10 min after the final injection. The resulting nanoparticles were precipitated, washed and re-dispersed in 0.4 mL of cyclohexane. The preparation method for the $NaYF_4$:Er@$NaYF_4$ (14 nm) core–shell nanoparticles is similar, except that using 0.2 mL of as-synthesized $NaYF_4$:Er@$NaYF_4$ (7 nm) core–shell nanoparticles as the seed, reducing OA/ODE amount to half and adjusting the injection repetition to 30 times. And finally re-disperse the nanoparticles in 0.2 mL of cyclohexane.

Synthesis of ligand-free nanoparticles. The as-prepared core–shell nanoparticles in cyclohexane (4 mL) were extracted and re-dispersed in 8 mL HCl solutions (0.1 M). The slurry solution was then sonicated at room temperature for 1 h and kept still for overnight in order to remove the surface oleate ligands. After the reaction, the ligand layer was discarded, and the nanoparticles were collected *via* centrifugation at 14000 rpm for 30 min and re-dispersed in ethanol. The washing process was repeated twice and the ligand-free nanoparticles were finally re-dispersed in 4 mL ethanol.

Synthesis of $NaErF_4$ microrods. The $NaErF_4$:Lu (20%) microrods were prepared via a hydrothermal method. Typically, $Er(CH_3CO_2)_3$ (1.6 mL, 0.2 M), $Lu(CH_3CO_2)_3$ (0.4 mL, 0.2 M) and trisodium (1.6 mL, 0.3 M) was added to 15 mL of deionized water under vigorous stirring to form a milky suspension. The resulting suspension was then transferred to a 30 mL Teflon-lined autoclave and heated to 200 ^oC for 36 h. The product was collected by centrifugation, washed with deionized water and ethanol several times, and finally dispersed in 10 mL ethanol.

Materials characterization. Powder X-ray diffraction (XRD) analysis was performed on a Bruker AXS D2 phaser with a graphite-monochro-matized Cu Kα radiation (λ = 1.5406 Å). Transmission electron microscopy (TEM) and high-resolution transmission electron microscopy (HRTEM) images were taken on a JEOL JEM 2100F transmission electron microscope at an acceleration voltage of 200 kV. Scanning electron microscopy (SEM) imaging of SU-8 pattern on the microring resonator was performed using a Hitachi S-4800 field emission SEM. Before SEM imaging, the surface of the device was coated with a gold layer to prevent surface charging. The luminescence spectra in the visible range were recorded with a Hitachi F-4600 spectrophotometer, in conjunction with a 980 or 1532 nm diode laser as the excitation source. The luminescence spectra in the NIR range and decays of cyclohexane dispersion of nanoparticle were acquired on an Edinburgh FLSP920 spectrometer equipped with a 808, 980, or 1532 nm pulsed laser and a nitrogen-cooled NIR photomultiplier rod (Hamamatsu R5509-72). Optical micrographs of all the devices were recorded with an advanced research microscope (ECLIPSE Ni-U, Nikon). All measurements were performed at room temperature. The Input−Through and Input−Drop port transfer function of the microring resonator was measured by the integration of a tunable laser, polarization synthesizer and optical power meter all connected with a Keysight Photonic Application Suite.

Fabrication of integrated optical circuit excitation platform. The integrated optical circuit excitation platform was fabricated from a high refractive index glass. The waveguide core of both the microring resonator and the waveguide loop are made of low-loss, high index doped silica glass that is semi-buried within a SiO_2. The waveguide dimensions and refractive indices are 1.5 μm x 1.5 μm and 2.0 μm x 2.0 μm, and n=1.70 and n=1.60, for the microring resonator and the waveguide loop respectively. The bus waveguide that couples to the ring waveguide is 0.9 μm x 1.5 μm. In the fabrication, high-index silica-glass films were first deposited using standard chemical vapour deposition. Subsequently waveguides were formed using photolithography and reactive ion etching, producing extremely smooth sidewalls. The waveguides were then buried in fused silica glass. To allow strong interaction between the nanoparticles and the optical fields in the waveguide, the top cladding of the device was removed by chemical-mechanical polishing to the top of the core waveguide. Unlike the devices reported in ref. [2-6], the devices reported here do not have a mode-transformer as the chemical-mechanical polishing process does not allow their formation, resulted with an approximately 10

dB addition coupling loss than that reported in ref. [2]. We reduced the radius of the microring resonator from 135 μm (FSR=200 GHz) to 47 μm (FSR=573 GHz) to further localize the excitation from the ring surface.

Excitation of $NaYF_4$:Er@$NaYF_4$ nanoparticles with the integrated optical circuit platform. The nanoparticles in an ethanol dispersion was drop cast on the surface of the pigtailed device and allow the full evaporation of ethanol. The excitation energy pumped by a tunable laser (81960A, Agilent.) first goes through a mini manual variable attenuator (Operating center wavelength at 1550 nm, Advanced Fiber Resources) to lower the excitation power and then a polarization controller (ProtoDel) was used to tune the polarization of excitation light. A 99:1 coupler was then utilized to assist reading 1% of the input power at 1550 nm before the laser finally arrived at the input port of microring resonator. To make sure that as much as possible of laser was coupled into microring resonator, Drop and Through ports were then monitored by using another power meters at 1550 nm (**Figure 4a**). The wavelength and polarization of the input laser were slowly adjusted until the power at the Drop port was maximized and the power at the Through port was minimized.

Experimental procedure for overlaying polymer waveguide on the microring resonator. 20 μL of $NaErF_4$@$NaYF_4$ nanoparticles in ethanol dispersion (0.01 M) was first drop-casted on the microring-resonator. After the ethanol was evaporated, a 2 μm thick SU-8 2002 was spin-coated on the device followed by baking at 90 ^{o}C for 10 min. The device was then placed on an alignment stage to align the input/output fiber array with the device. To align the wavelength and polarization of the laser with the microring resonator, we first use a very low input laser power and slowly tune the wavelength as well as adjust the polarization controller until power at the drop port is maximized. Using a very low laser power for finding the resonance wavelength was to avoid too much light exposure to the straight bus waveguide and microring resonator at this stage. Once the resonance and polarization are aligned between the input and the resonator, the laser was then increased to 20 μW to start the curing process. After 10 min the device was removed from the alignment stage and transferred to a 90 ^{o}C hotplate for 10 min of post-exposure bake procedure. After the device was cooled down to room temperature, then remove the unexposed part of SU-8 by immersing the device into the SU-8 developer for 1 min and finally resined in water.

Fabrication and excitation of upconversion microdisk. The upconversion microdisk was fabricated through a sequence of photolithography, inductively coupled plasma (ICP) etching, photoresist removing and spin coating.[7] Typically, the oxidized silicon wafer was first cleaned by acetone, isopropanol and deionized water. Then the photoresist (AZ2020, Microchem) was spin-coated onto the wafer, followed by a standard photolithography protocol to pattern microstructure within the photoresist. The microstructure was then transferred onto the silica by performing the ICP etching procedure with C_4F_8 as the anisotropic etching gas. Subsequently, the photoresist was thoroughly removed by a soaking process. And finally, the mixture of 2.9 wt% $NaErF_4$@$NaYF_4$ nanoparticles and silica resin was spin-coating onto the preformed SiO_2 substrate to form the upconversion microdisk.

Calculation setup for relative oscillator strength. For calculating the energy states, we used an efficient formulation of time-dependent linear response density functional theory for the use within the plane wave basis set framework developed by Hutter.[8] Regarding the time-dependent density functional theory calculation functionalities, the related modules and codes have been recently implemented within the density functional perturbation theory (DFPT) framework within CASTEP package.[9] Under the excitation theoretical calculation, consensuses have been approached that the excitation light source can usually be simulated as external frequency-dependent electric field and the electronic orbital level-variation are described by the electronic density response. Therefore, this approach can effectively compute all the corresponding response by valence electrons regarding the targeted external electric field of a set frequency.

Different from the Gaussian-based computational manner, we imported the theoretical method introduced by the pioneering work of Hutter.[8] This can reach computationally more efficient and the non-local pseudopotential technique can better approach the scaling linearly for the size with accuracy maintained. Moreover, Hutter's method avoids the transformation of the Kohn-Sham orbital matrix into the canonical basis and projects the orbitals into occupied and virtual parts. Further applied Tamm-Dancoff approximation (TDA)[10] with Lagrangian formulation, the geometry optimization is feasibly conducted under the excited states.

$$\left(H^{(0)} - \varepsilon_i\right)\left|\Phi_i^{(\pm)}\right\rangle + P_c \delta V_{SCF}(\pm\omega)\left|\Phi_i^{(0)}\right\rangle = \mp\omega\left|\Phi_i^{(\pm)}\right\rangle \quad (1)$$

The above equation is the *equation 17* of the Hutter's theoretical development work.[8] In this equation, the term $H^{(0)}$ is the ground state total energy (described by Kohn-Sham orbitals) Hamiltonian of the targeted system, ε_i represents the ground state eigenvalue for the band index i (described under Kohn-Sham orbitals). The P_c is the projector on the subspace of the unperturbed empty states, and the operators $\left|\Phi_i^{(\pm)}\right\rangle$ are the electronic response wavefunctions to the external electric-field perturbation of a given frequency ω. The wavefunction $\left|\Phi_i^{(0)}\right\rangle$ describes the orthogonal occupied Kohn Sham orbitals at the ground state and $V^{(1)}(\pm\omega)$ is the response potential, containing contributions from the correlation-free electron-electron Hartree potential, non-local exchange-correlation, and the external electric field perturbation contributions. Also, combined in the Tamm-Dancoff approximation (TDA), some necessary simplification treatments from the Hutter's theory are required, such as the occupied-to-virtual overlapping contributions are disregarded, while the virtual-occupied ones are still kept. This results in the term $\left|\Phi_i^{(+)}\right\rangle = 0$, and the theme equation is updated as:

$$\left(H^{(0)} - \varepsilon_i\right)\left|\Phi_i^{(-)}\right\rangle + P_c \delta V^{(1)}(-\omega)\left|\Phi_i^{(0)}\right\rangle = \omega\left|\Phi_i^{(-)}\right\rangle \tag{2}$$

The Eq. 2 is the essential equation written in the coding implemented for the electric-field-based (simplified laser light source) response in the CASTEP package. Moreover, our recent efforts have been startup for developing the tunable ω of the chosen external frequency and meanwhile the implementation of the double-laser-light-source excitations referred to the theoretical works of Lin et al.[11-12]

It is also worth to mention the advantage of this method. To model the overall excited state levels for the targeted nanoparticle structural models, accurate calculations have met the bottleneck limited by the computation-loading based on the current method. In detail, the small solid particle systems usually contain over thousand electronic states that urges tremendous computation cost over several hundreds of gigabytes in memory size for data input/output (I/O), by using conventionally improved wavefunction-based method.

According to the above detail explanations on the theoretical fundaments, the relative oscillator strength (ROS) that we used here have been further derived from the as-calculated

first-order electric-dipole transition based excited levels by the Hutter's method where the time-dependent density functional theory (TD-DFT) modules have been actualized within CASTEP package.

During the performed excited state calculation, we chose the two-electron based Tamm-Dancoff approximation imported from self-consistently corrected ground state wavefunctions.[10] To guarantee the stability of the electronic diagonalization process, currently we only consider using the block-davidson solver. Regarding different doped nanoparticle modeling system, the hexagonal phase β-$NaYF_4$ comprising different amount of Er^{3+} dopants (12.5% to 100%) were examined by a series of our developed hybrid TD-DFT calculations. The calculated excitation energies can be used for post-processing to determine the locations of the absorption peaks in the optical spectrum, which can be more accurate than Kohn-Sham excitation energies. For determining the excitation direction and specific orbital-to-orbital one-to-one transition, the post-analysis of the electronic excited band overlapping with calculated probability are used, which have been comprehensively yielded after the excited states go throughout all of the real-occupied and virtual un-occupied states with traversing all of the electronic band index during the TDDFT calculation.

The ground state wavefunction and the related electronic structure were calculated by simplified rotational invariant DFT+U method using the CASTEP source codes.[9] Hexagonal lattice with the $P\overline{6}$ space group is modeled for β-phase $NaREF_4$ (RE=Y and Er). The Na, Y, Er, and F norm-conserving pseudopotentials are generated using the OPIUM code in the Kleinman-Bylander projector form,[13] and the non-linear partial core correction.[14] A scalar relativistic averaging scheme[15] were applied to treat the spin-orbital coupling effect. In particular, we treat the (4*f*, 5*s*, 5*p*, 5*d*, 6*s*) states as the valence states of Er atom. The RRKJ method was chosen for the optimization of the pseudopotentials.[16]

For more accurate calculations of the excited electronic states in β-$NaREF_4$ (RE=Y and Er), we used the self-consistent determination for the U correction on the localized 4f orbitals to correct the on-site Coulomb energy of the electron spurious self-energy. This is a two-way crossover linear response that generally searches for the optimal Hubbard U parameter to minimize the residue of the counteracting electronic self-energy and the orbital relaxation in the excited states. In order to obtain accurate orbital eigenvalues for electronic structures and transition levels, we have established an algorithm to determine the on-site electronic self-energy

and related wavefunction relaxation in the semicore d or f orbitals in heavy elements with mixed valence.[17-26] The algorithm is based on ab-initio two-way crossover linear response searching calculations by two different sets of functionally compiled CASTEP-17 developing source codes.[22,27-28] The detail process is described in a previous work,[24] and the schematic theoretical determination process is demonstrated in ref. [29]. With the self-consistently determination process, the on-site Hubbard U parameters for 4f of Er, 4d of Y, and different 2p of F-sites were obtained respectively.

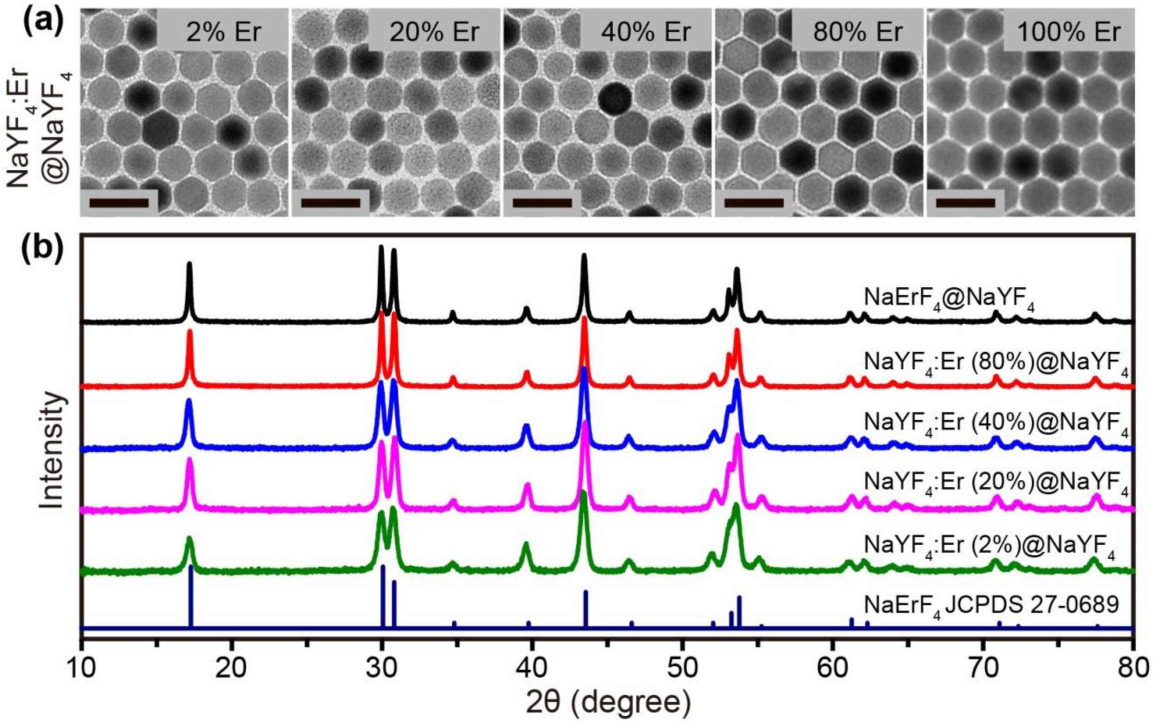

Supplementary Figure 1. Layer-by-layer growth of the $NaYF_4$:Er@$NaYF_4$ core–shell nanoparticles. (a) TEM images and (b) XRD spectra of the nanoparticles doped with different Er^{3+} concentration. Scale bars for TEM images are 50 nm. The line spectrum in (b) is literature data for hexagonal-phase $NaErF_4$ (JCPDS 27-0689).

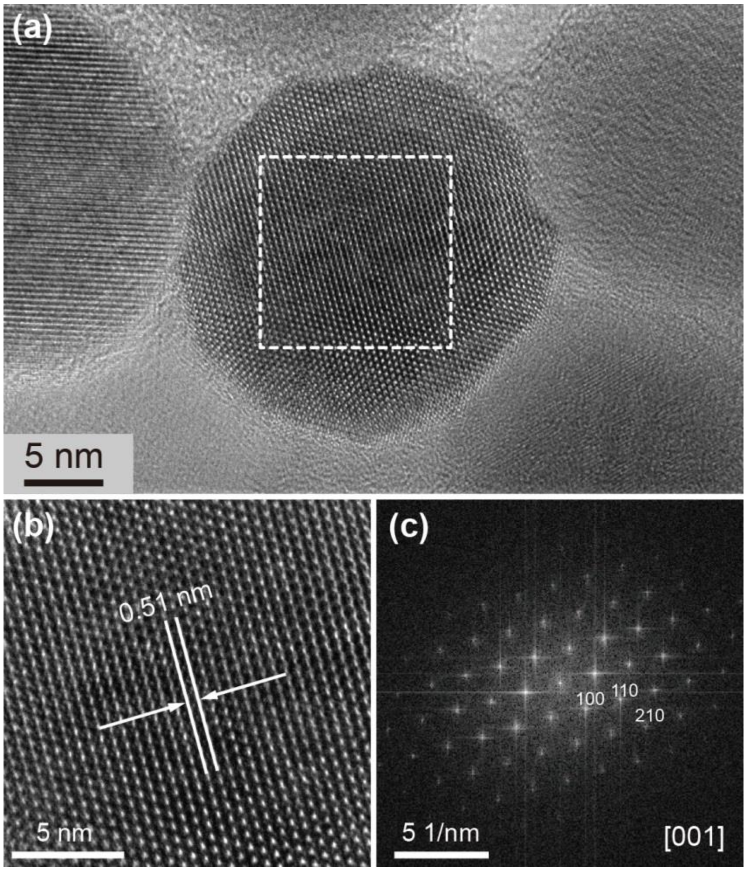

Supplementary Figure 2. High-resolution TEM characterization of the $NaErF_4$@$NaYF_4$ nanoparticles. (a) High-resolution TEM image revealing single-crystalline nature of the nanoparticles. (b) An enlarged view of the selected area in (a), indicated by a white box, showing well-defined lattice fringes with an observed *d* spacing of 0.51 nm. (c) Fourier transform diffraction patterns of the region indicated in (a), indicating the hexagonal phase of the nanoparticle.

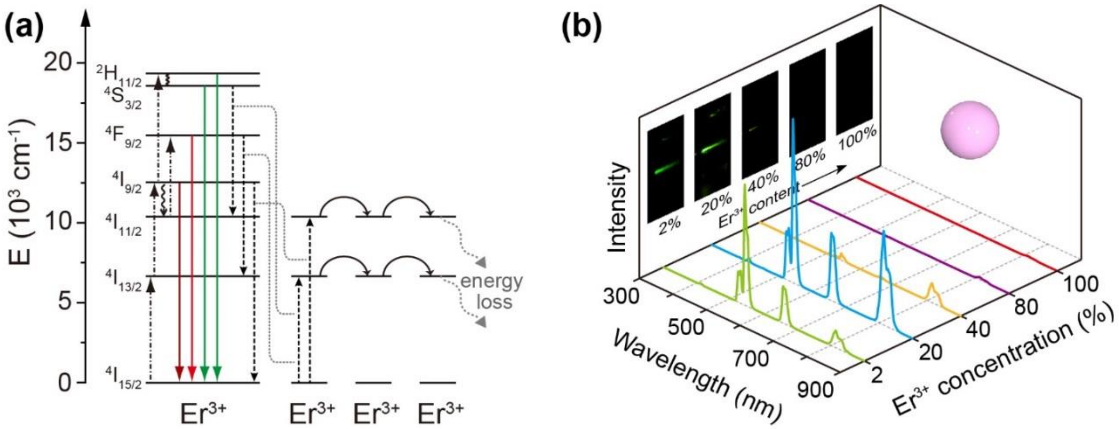

Supplementary Figure 3. Concentration quenching in the $NaYF_4$:Er core nanoparticles. (a) Proposed energy transfer mechanism in $NaErF_4$ core nanoparticles under 1532 nm excitation. (b) Emission spectra of $NaYF_4$:Er (2−100%) core nanoparticles in cyclohexane dispersions (0.2 M) as a function of Er^{3+} concentration. The samples were excited with a 1532 nm CW diode laser at a power density of 21 W cm^{-2}. Inset: luminescence photographs of the corresponding nanoparticle colloids.

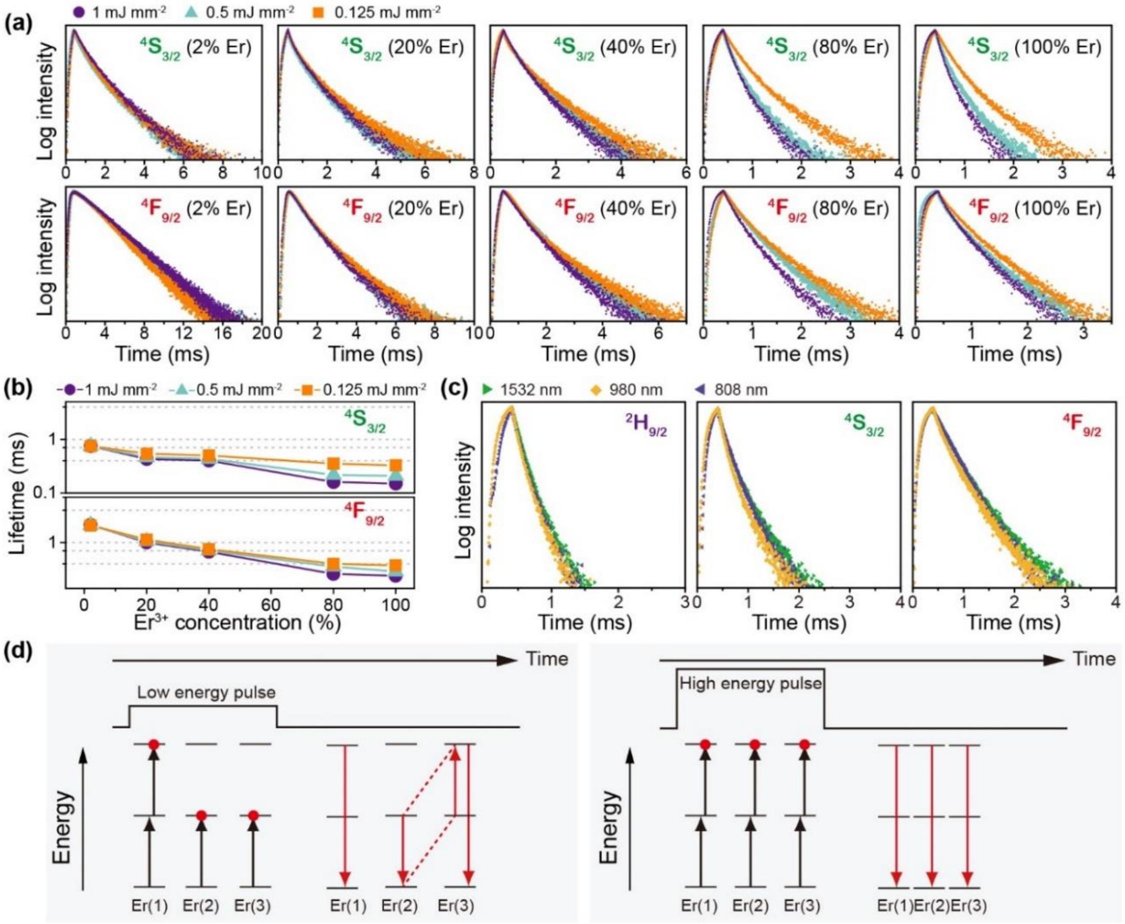

Supplementary Figure 4. The effect of excitation conditions on measured time decay. (a) Decay curves and (b) calculated lifetime of $^4S_{3/2}$ and $^4F_{9/2}$ states of Er^{3+} in the $NaYF_4$:Er@$NaYF_4$ core–shell nanoparticles under excitation of 980 nm at different powers density. The results show strong dependence of measured lifetimes on excitation power density, especially for samples comprising high concentrations of Er^{3+}. (c) The decay curves of various excited states of Er^{3+} in the $NaErF_4$@$NaYF_4$ core–shell nanoparticles under excitations of different wavelengths, showing the marginal dependence of Er^{3+} lifetimes on wavelength of NIR excitation. The energy density for different excitation wavelengths was all set at 1 mJ mm^{-2}. (d) Proposed mechanism for the excitation power dependent decay behavior. Low-power excitation results in a large population in the low-lying intermediate state. After cutoff of the excitation, energy transfer upconversion replenishes the population at the high-lying emitting state, which lengthens the measured lifetimes. At high excitation powers, the population in the high-lying emitting states is mostly established by the excitation pulse and thus the measured lifetimes are shortened. The similar decay curves under different excitation wavelength in (c) are ascribed to close population density in the intermediate states induced by the excitation pulses. To examine the changes in lifetimes of Er^{3+} as a function of dopant concentration, we used a high energy pulse (1 mJ mm^{-2}) to minimize the interference of energy transfer to the measured decay curves and to enhance the signal-to-noise ratio.

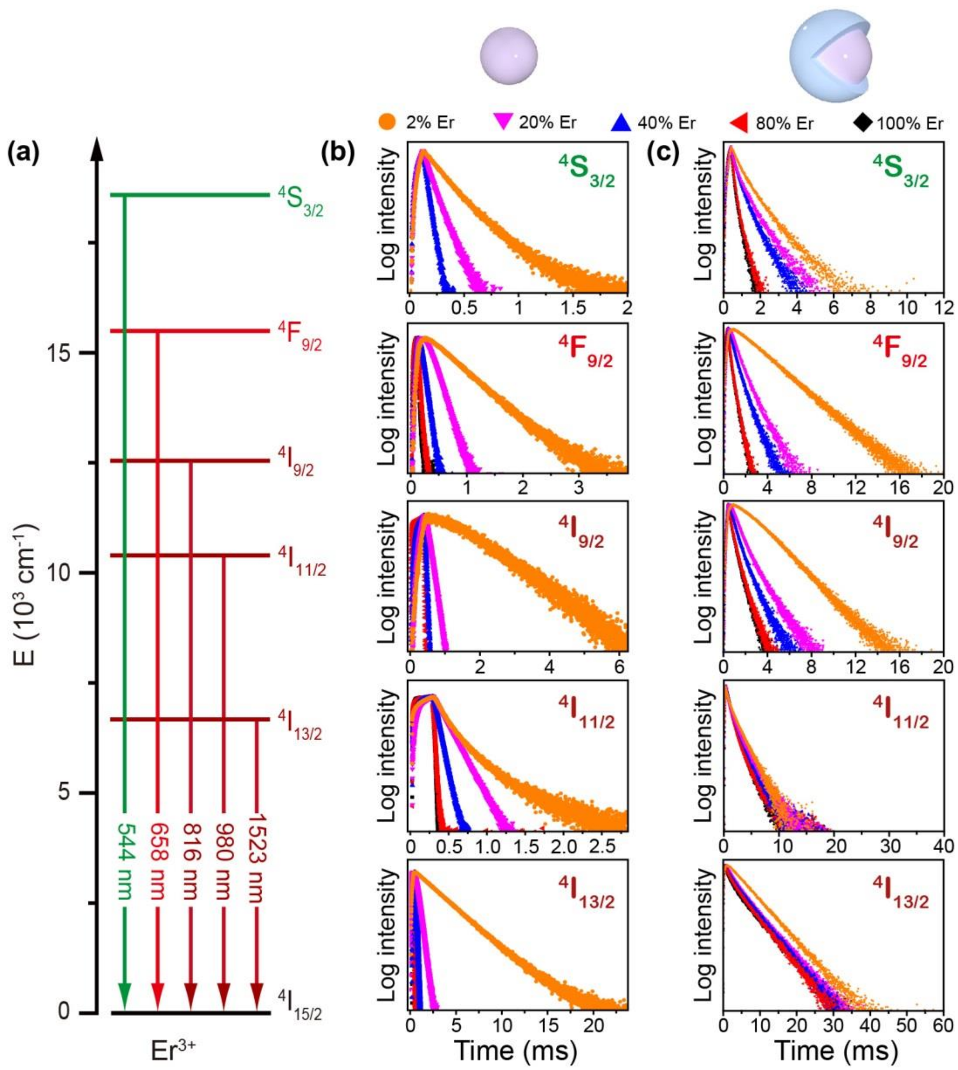

Supplementary Figure 5. Comparative time decay study of the $NaYF_4$:Er core and the $NaYF_4$:Er@ $NaYF_4$ core–shell nanoparticles. (a) Simplified energy level structure showing the excited states of Er^{3+} being investigated. (b, c) The decay curves of different excited states of Er^{3+} obtained from the core and core–shell nanoparticles, respectively. The decay curves of the $^4S_{3/2}$, $^4F_{9/2}$, $^4I_{9/2}$, $^4I_{13/2}$ states were measured under excitation of a 980 nm pulsed laser at am energy density of 1 mJ mm^{-2}. The decay curves of the $^4I_{11/2}$ state were measured under excitation of an 808 nm pulsed laser at the same pulse energy density.

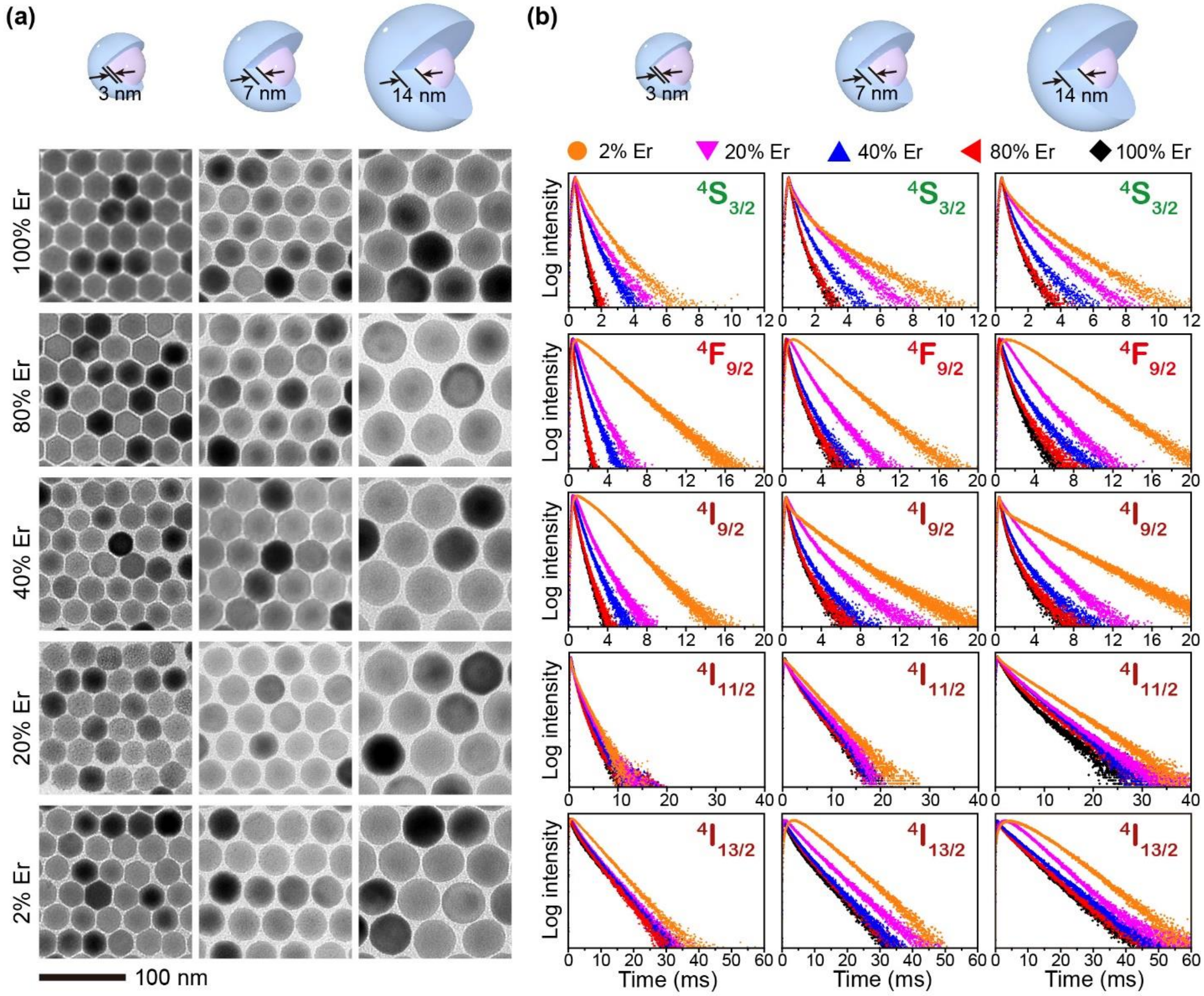

Supplementary Figure 6. Layer-by-layer growth of the $NaYF_4$:Er@$NaYF_4$ core–shell nanoparticles with various shell thicknesses. (a) The shell thickness was tuned by adding different amount of shell precursor through a successive hot-injection method. The mean shell thickness of the final $NaYF_4$:Er@$NaYF_4$ core–shell nanoparticles is 3 nm, 7 nm, and 14 nm, respectively. (b) The decay curves of different excited states of Er^{3+} obtained from the core–shell nanoparticles with various shell thicknesses. The decay curves of the $^4S_{3/2}$, $^4F_{9/2}$, $^4I_{9/2}$, $^4I_{13/2}$ states were measured under excitation of a 980 nm pulsed laser at an energy density of 1 mJ mm^{-2}. The decay curves of the $^4I_{11/2}$ state were measured under excitation of an 808 nm pulsed laser at the same energy density. Although thicker shells provided better protection to the core nanoparticles, the trends of lifetime evolution as the dopant concentration increases are essentially the same.

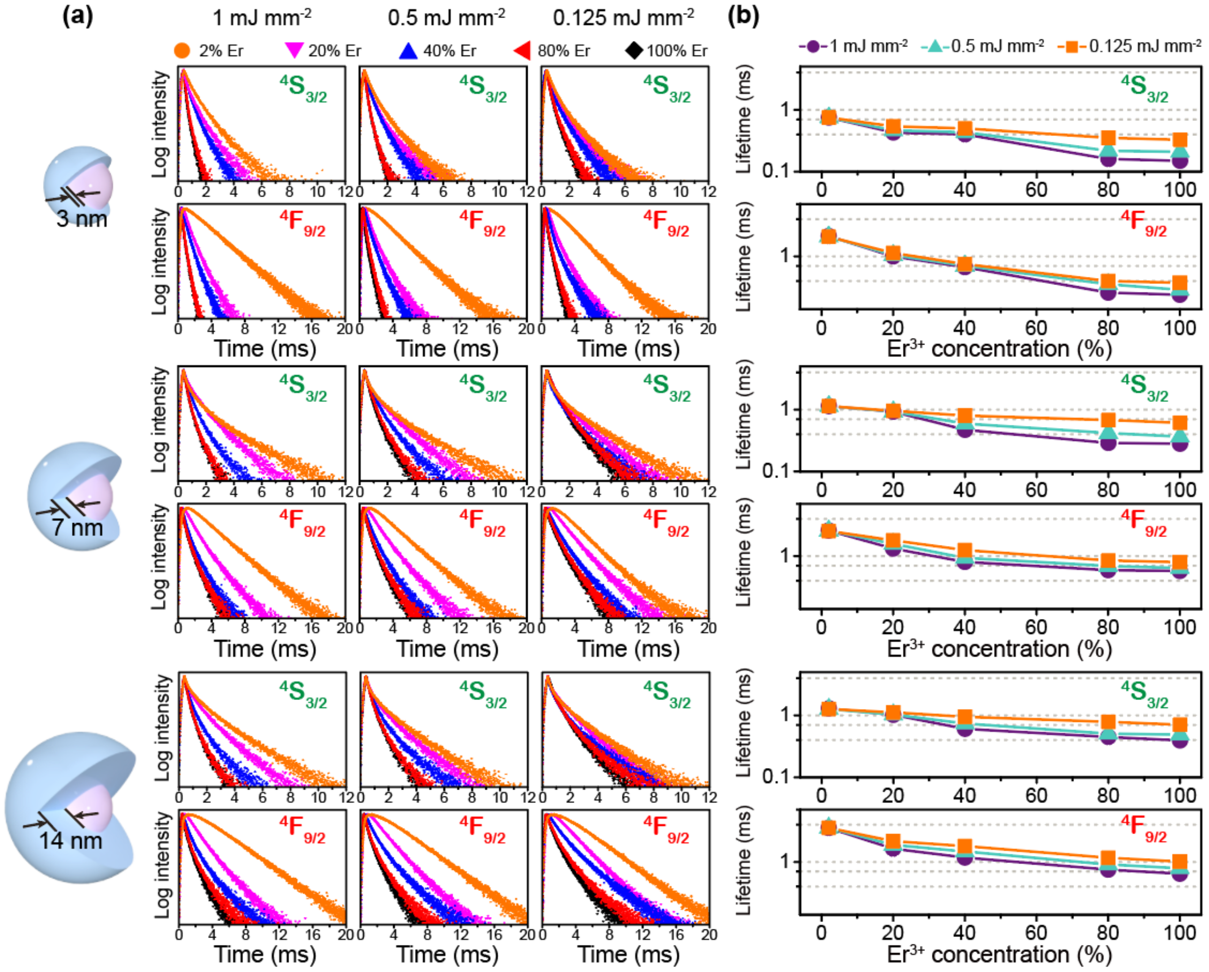

Supplementary Figure 7. The effect of excitation power on measured time decay. (a) Decay curves and (b) calculated lifetimes of various excited states of Er^{3+} in the $NaYF_4$:Er@$NaYF_4$ core–shell nanoparticles of varying shell thickness under excitation of 980 nm at different energy densities. Decay measurements detected a shortening of Er^{3+} lifetime for each excited state with increasing excitation power density. Lifetime of nanoparticle containing higher Er^{3+} concentration is more susceptible to excitation power variance. These behaviors apply for all sets of nanoparticles with various thickness of shell layers. The lifetime power dependent behavior is explained in Figure S4d. To study the effect of Er^{3+} concentration on the time decay, a high energy pulse (1 mJ mm^{-2}) was thus consistently used to minimize the interference of energy transfer to the measured decay curves and to enhance the signal-to-noise ratio.

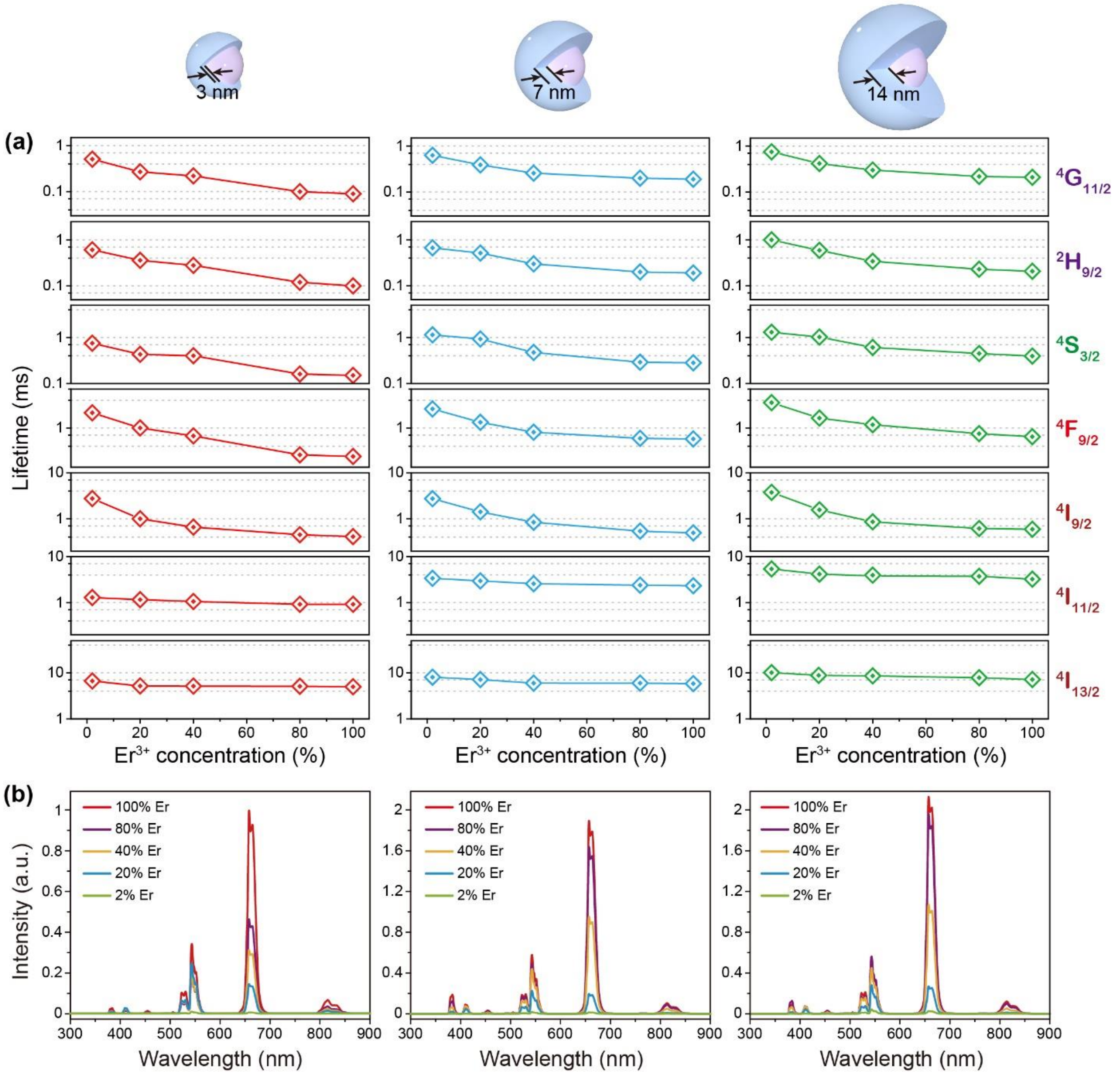

Supplementary Figure 8. Comparative spectroscopic investigations of the NaYF$_4$:Er@NaYF$_4$ nanoparticles with various shell thicknesses. (a) Lifetimes of different excited states Er^{3+} as a function of the dopant concentration in the NaYF$_4$:Er (2−100%)@NaYF$_4$ core−shell nanoparticles with a mean shell thickness of 3 nm, 7 nm, and 14 nm, respectively. Lifetime of $^4I_{11/2}$ state was measured under excitation of an 808 nm pulsed laser at an energy density of 1 mJ mm^{-2}. Lifetimes for the other energy states were measured under excitation of a 980 nm pulsed laser at the same energy density. (b) Emission spectra of NaYF$_4$:Er (2−100%)@NaYF$_4$ nanoparticles in cyclohexane dispersions (0.01 M) as a function of Er^{3+} concentration in core−shell nanoparticle with a mean shell thickness of 3 nm, 7 nm, and 14 nm, respectively. All emission spectra were recorded under excitation of a 1532 nm CW diode laser at a power density of 21 W cm^{-2}.

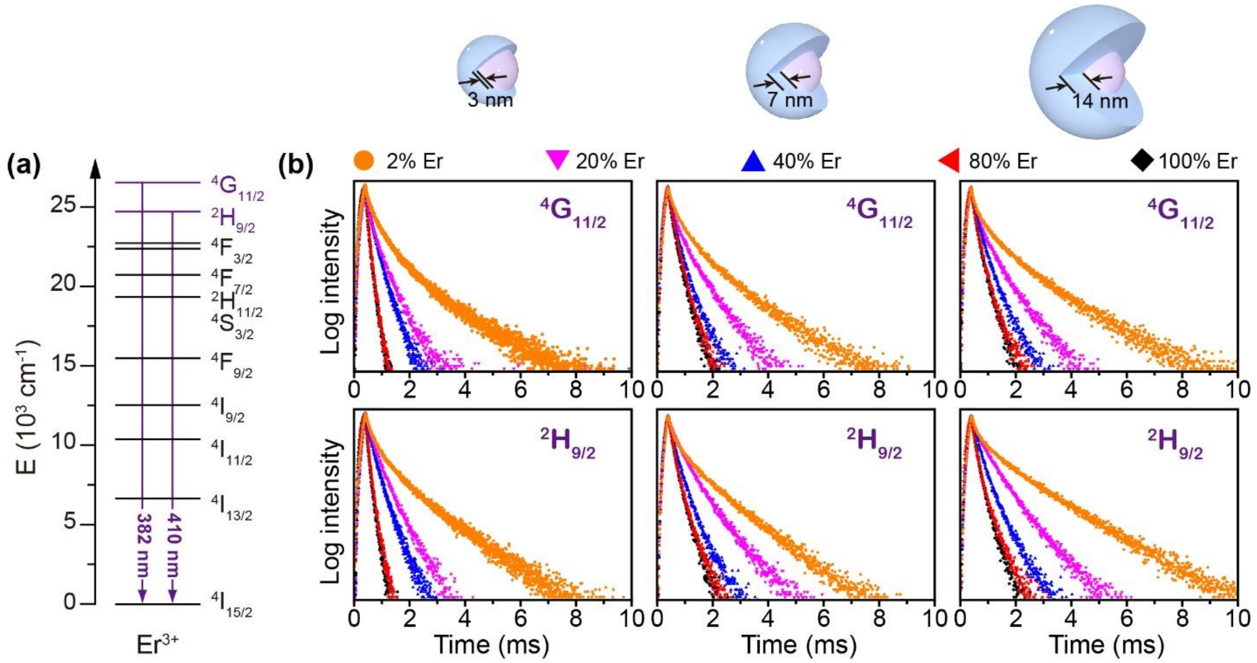

Supplementary Figure 9. Concentration quenching of high-lying emitting states in the $NaYF_4$:Er@$NaYF_4$ core–shell nanoparticles with various shell thicknesses. (a) Simplified energy level structure showing the emissions due to the $^4G_{11/2}$ and $^2H_{9/2}$ states of Er^{3+}. (b) The decay curves as a function of Er^{3+} concentrations for the $^4G_{11/2}$ and $^2H_{9/2}$ states for nanoparticles with a mean shell thickness of 3 nm, 7 nm, and 14 nm, respectively. Decay curves were measured under excitation of a 980 nm pulsed laser at an energy density of 1 mJ mm^{-2}. Note that the decay curves of the $^4G_{11/2}$ and $^2H_{9/2}$ states for the core nanoparticles could hardly be recorded due to the extremely low photon counts.

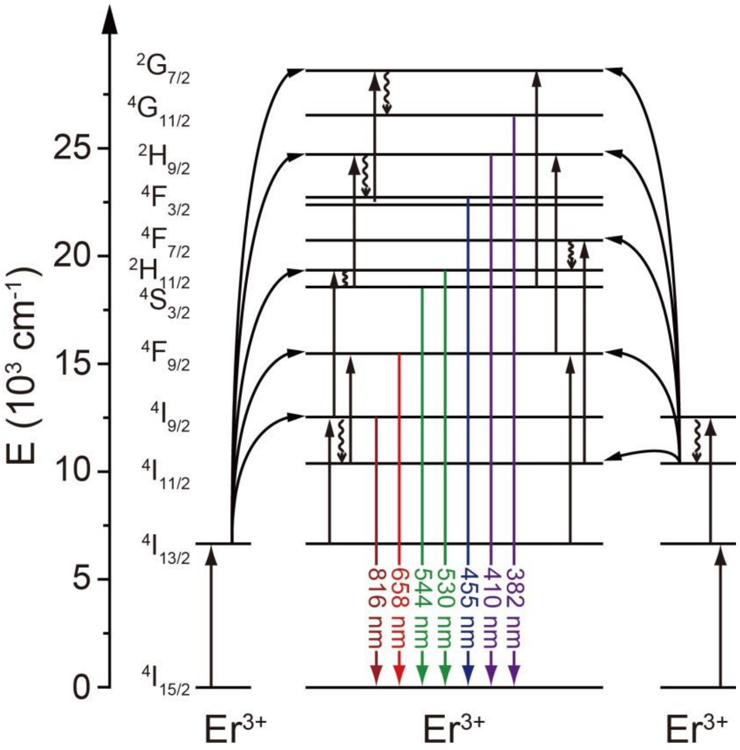

Supplementary Figure 10. Depletion of the $^4I_{13/2}$ and $^4I_{11/2}$ excited states of Er^{3+} by energy transfer upconversion. The simplified energy level structure illustrates the proposed energy transfer mechanism in the $NaErF_4$@$NaYF_4$ nanoparticles.

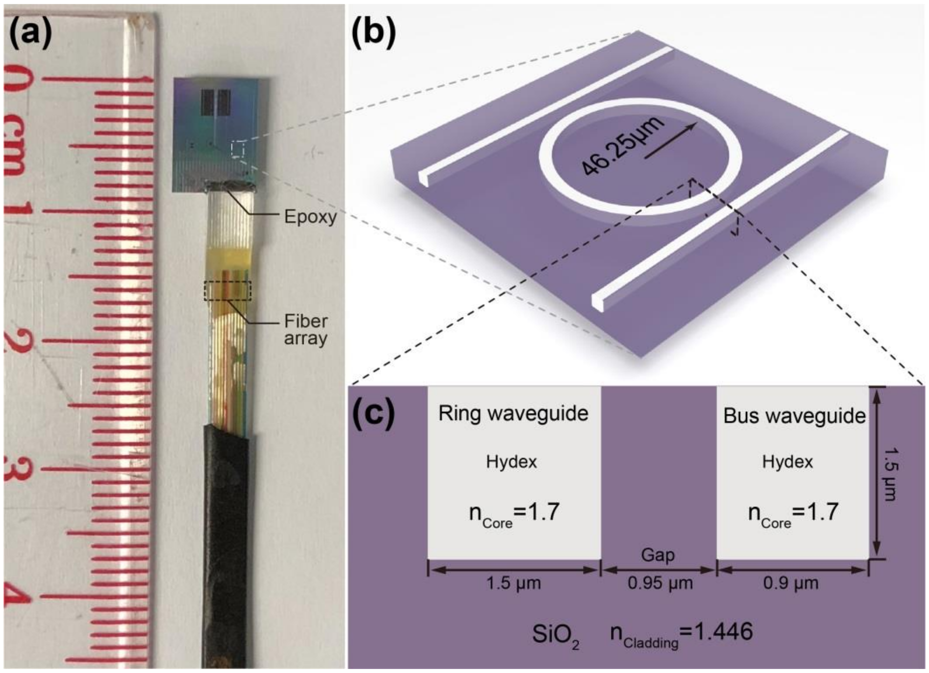

Supplementary Figure 11. The microring resonator. (a) The photograph of the device used in the experiment. (b, c) The geometry and dimension of the microring resonator. The waveguide (n=1.7) was embedded into the SiO_2 substrate (n=1.446) with the top surface exposed to the environment. Schematic is not drawn to scale.

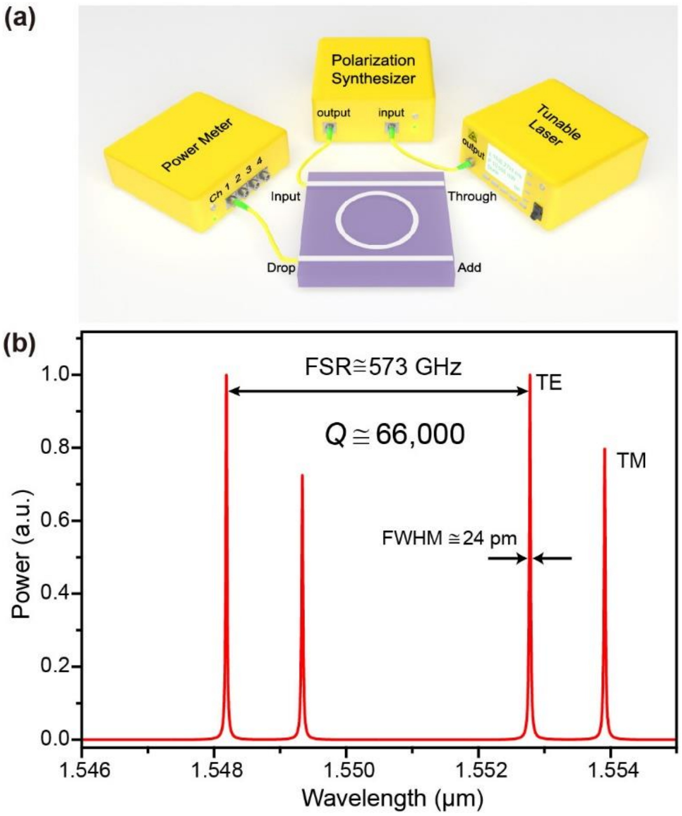

Supplementary Figure 12. Measurement of Input–Drop port transfer function. (a) The setup for the measurement. The IL/PDL measurement system (the Keysight Photonic Application Suite) was used to measure the optical responses of the microring resonator device. (b) Input–Drop port transfer function from λ = 1546 nm to 1555 nm for both transverse electric (TE) and magnetic (TM), showing a Q-factor of ~66000, a free-spectral range (FSR) of 573 GHz and a full-width at half-maximum (FWHM) of 24 pm.

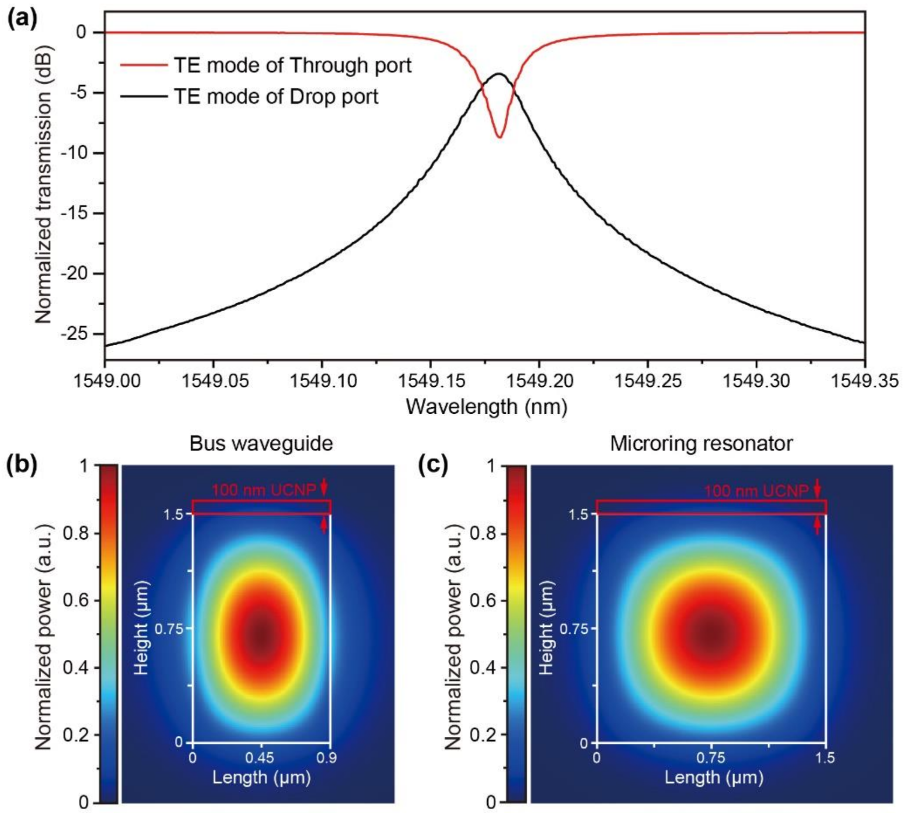

Supplementary Figure 13. Calculation of the excitation power density. (a). Input–Drop (black) and Input–Through (red) port transfer function from λ = 1549 nm to 1549.35 nm for transverse electric (TE), showing a full-width at half-maximum (FWHM) of 26.32 pm, *Q*-factor of 58859, a Finesse *F* of the microring resonator of 351. Accordingly, the intensity enhancement factor *B* by the microring resonator is calculated to be 23. Electrical field distribution of the TE polarized beam in (b) bus waveguide and (c) ring resonator. According to our simulation, 0.6% and 0.75% of total laser powers were received by the nanoparticles on top of the bus and ring waveguides, respectively. At an input laser power of 20 mW, the excitation power density of nanoparticle on the bus waveguide was calculated as (20 mW $\times$ 10^{-3} $\times$ 0.6%) / (0.9 μm $\times$ 100 nm $\times$ 10^{-11}) = 133 kW cm^{-2}. Similarly, the excitation power density of nanoparticles on the ring waveguide was calculated as (20 mW $\times$ 23 $\times$ 10^{-3} $\times$ 0.75%) / (1.5 μm $\times$ 100 nm $\times$ 10^{-11}) = 2300 kW cm^{-2}.

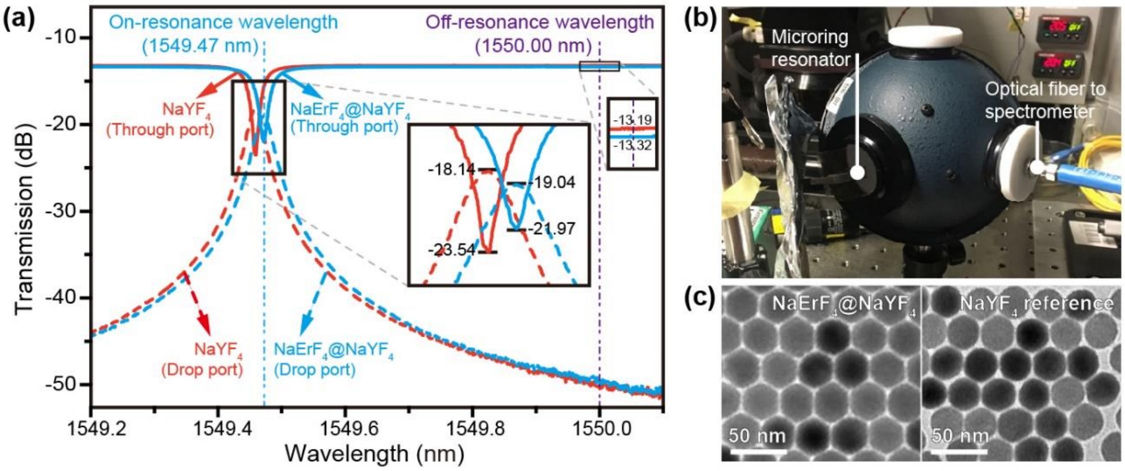

Supplementary Figure 14. Measurement of energy conversion efficiency of nanoparticles under microring resonator-assisted excitation. (a) Optical responses of the through and drop ports of the fiber coupled microring resonator coated with the reference $NaYF_4$ nanoparticles (red line) and upconversion $NaErF_4$@$NaYF_4$ nanoparticles (blue line). The measured on/off coupling loss of the reference $NaYF_4$ nanoparticles coated device is 13.19 dB, corresponds to per facet loss of 6.60 dB neglecting the propagation loss in the waveguide. In the measurement, a tunable laser with a fixed power of 91.3 mW was launched into the input port, corresponded to 20.0 mW coupled into the waveguide after considered the per facet loss. The power absorbed by the upconversion nanoparticles (P_{smp}) was determined from the comparison of the total power from the through and drop ports between the reference T_{ref} and the upconversion sample T_{smp}. For the reference $NaYF_4$ nanoparticles, the transmitted power at the through and drop ports are -23.54 dB and -18.14 dB when the device is on resonance at 1549.46 nm, which corresponds to 1.85 mW and 6.40 mW at the input power of 20.0 mW for a total power $T_{ref}^{on} = 8.25\text{ mW}$. Similarly, for upconversion $NaErF_4$@$NaYF_4$ nanoparticles, the total output power $T_{smp}^{on} = 7.85\text{ mW}$. The absorption of upconversion nanoparticles was then can be calculated to 400 μW with the ring is on-resonance. At off-resonance there is negligible power at the drop ports and only the through port powers were considered. From the measured transmission of -13.19 dB and -13.32 dB for reference and upconversion nanoparticle at 1550 nm. The output powers are $T_{ref}^{off} = 20\text{ mW}$ and $T_{smp}^{off} = 19.41\text{ mW}$ for reference and upconversion nanoparticles, respectively. The absorption of upconversion nanoparticles was then can be calculated to 590 μW at off resonance. (b) The emission is collected by an integrating sphere (Labsphere) of 3-inch in diameter and detected by an Ocean Optics USB 2000 spectrometer (spectral range 180–880 nm). The recorded spectra were calibrated with a standard halogen lamp and converted into spectral power distribution. The total radiant fluxes (Φ) of device were determined to be 6.6 μW and 20.1 μW at the off- and on-resonance wavelengths (1550.00 and 1549.47 nm), respectively. Accordingly, the off- and on-resonance energy conversion efficiencies (η = Φ / P_{smp}) are 6.6 μW / 590 μW = 1.1% and 20.1 μW / 400 μW = 5.0%. (c) TEM micrographs showing close size of the $NaErF_4$@$NaYF_4$ upconversion nanoparticles and the $NaYF_4$ reference nanoparticles.

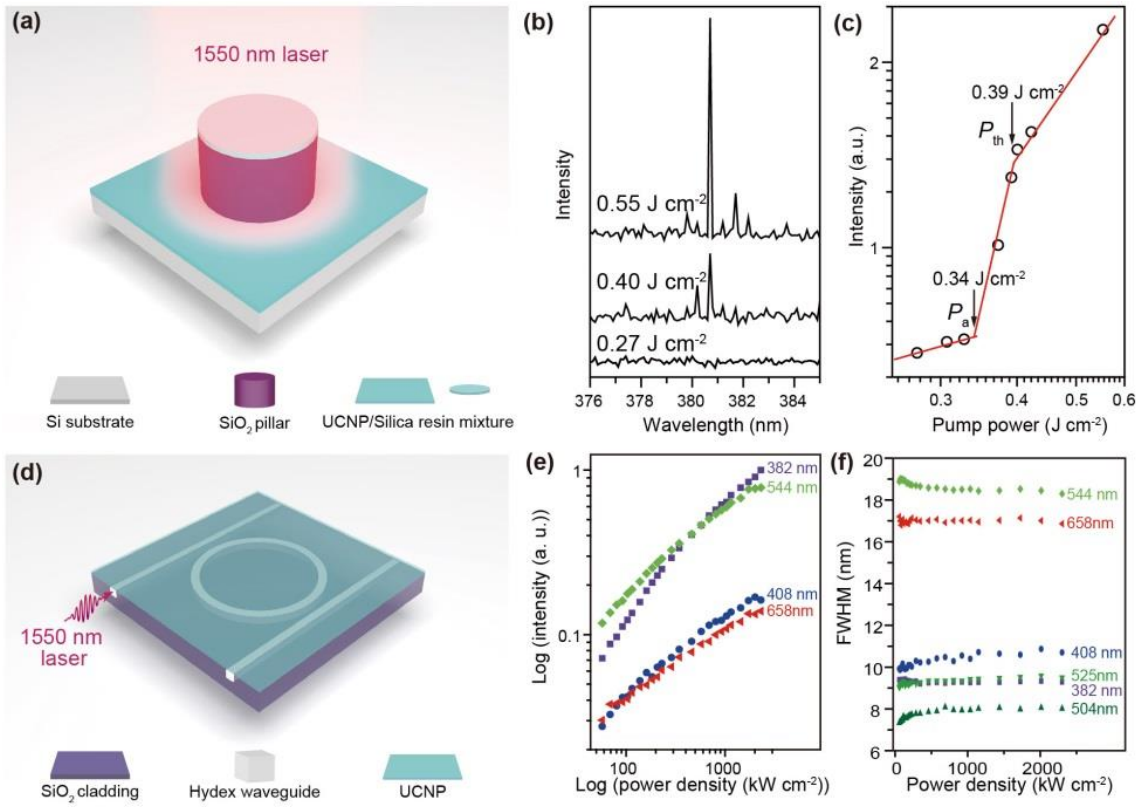

Supplementary Figure 15. Upconversion lasing from the $NaErF_4$@$NaYF_4$ nanoparticles. (a-c) Schematics and laser characteristics of an upconversion nanoparticle (UCNP)-based microdisk (diameter: 60 μm, thickness: 300 nm) at the top of a SiO_2 micropillar (thickness: 3 μm). The UCNP-based microdisk confines the emission in the radial direction due to continuous total internal reflection. (d-f) Optical measurements on the upconversion nanoparticles (UCNP) deposited on the microring resonator. No clear narrowing of the emission peaks or leap of emission intensity were observed, suggesting the absence of amplified spontaneous emissions (ASE). The results were ascribed to i) the large propagation loss of upconverted emission in the microring resonator and ii) the limited pumping flux available in the resonator (over high input power damages the epoxy at the fiber/device interface). Noted that the green emissions were deconvoluted into three peaks (504, 525 and 544 nm) for calculation of full width at half maximum (FWHM).

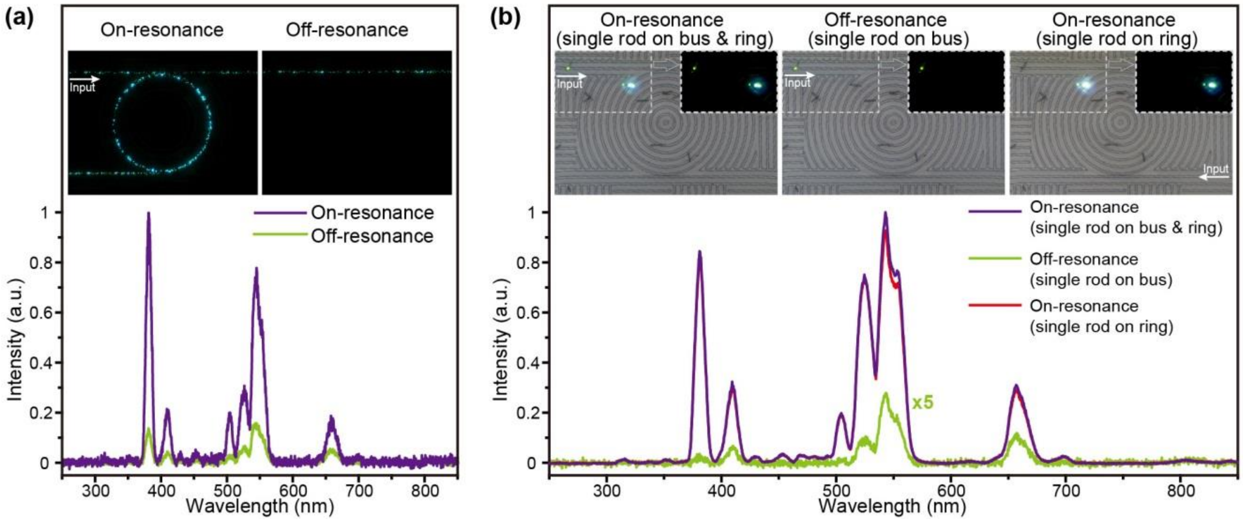

Supplementary Figure 16. Microring resonator-assisted excitation of few and single upconversion particles. (a) Emission spectra and optical micrographs recorded from a discontinuous layer of $NaErF_4$@$NaYF_4$ nanoparticles deposited on the microring resonator. (b) Emission spectra and optical micrographs recorded from single $NaErF_4$:Lu (20%) microrods on the microring resonator. Note that Lu^{3+} dopant was introduced to alleviate energy migration-induced concentration quenching by increasing the particle size and by diluting the erbium sublattice. The slightly weaker UV emission from the microrod with respect to that from the core–shell nanoparticles are due to: i) The microrod was lack of a surface protection layer. ii) The Er^{3+} concentration was lower in the microrod. iii) The microrod was less efficiently interacted with the evanescent field of waveguides due to the larger size.

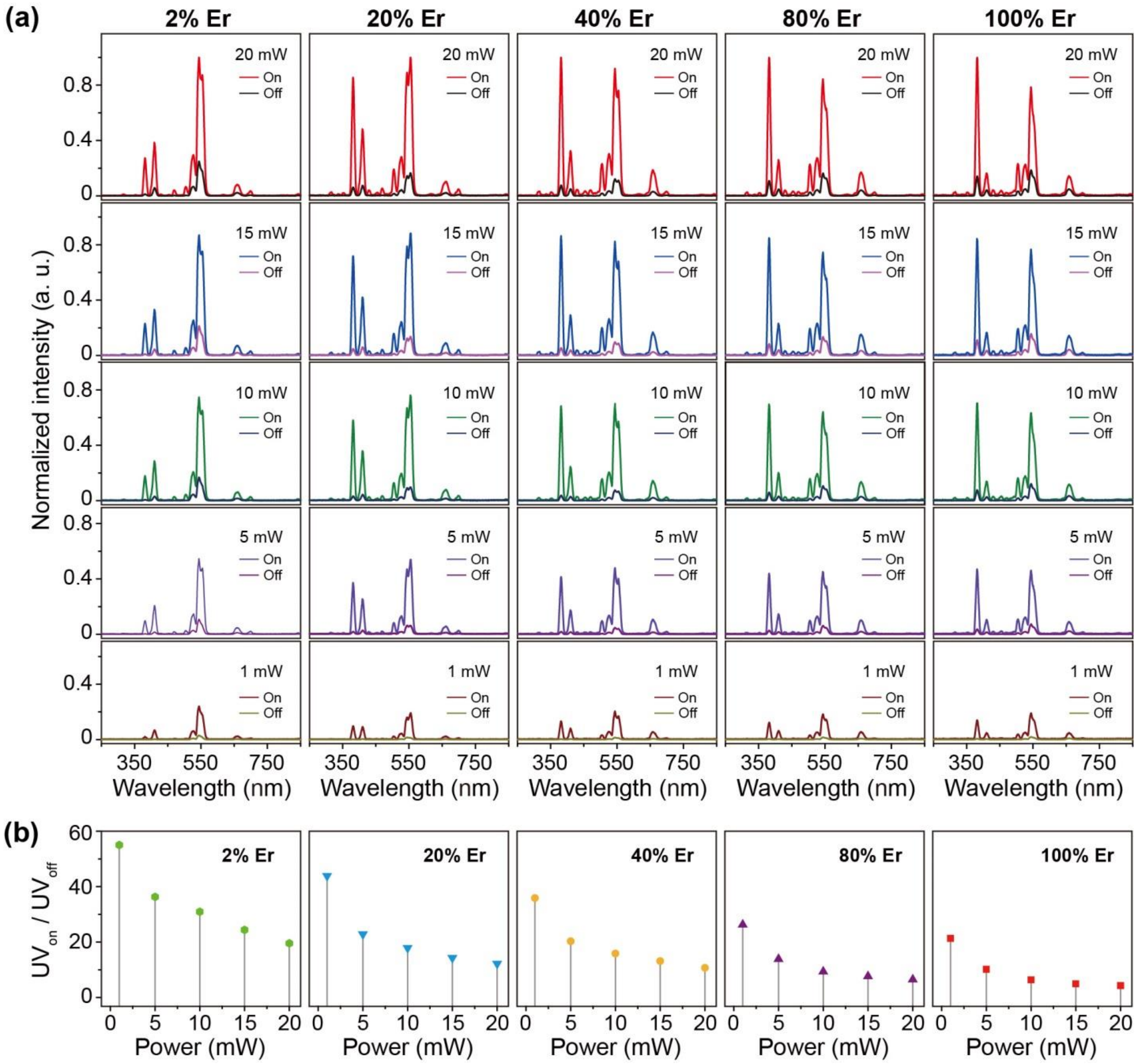

Supplementary Figure 17. Performance of the waveguide circuit in enhancing upconversion luminescence in nanoparticles of various compositions and at different input powers. (a) Emission spectra recorded in the on- and off-resonance states. Emission spectra for each sample were normalized to its maximum emission peak at 20 mW excitation. (b) The ratio of UV emission at 382 nm between the on and off states. In general, the enhancement effect weakens with increasing excitation power and Er^{3+} concentration due to saturation of upconversion emissions in the high-power regime. The results also show that a conventional waveguide excitation scheme needs to use ~20-fold higher excitation power than the microring resonator (i.e.; 20 mW *versus* 1 mW) to render the same emission profile.

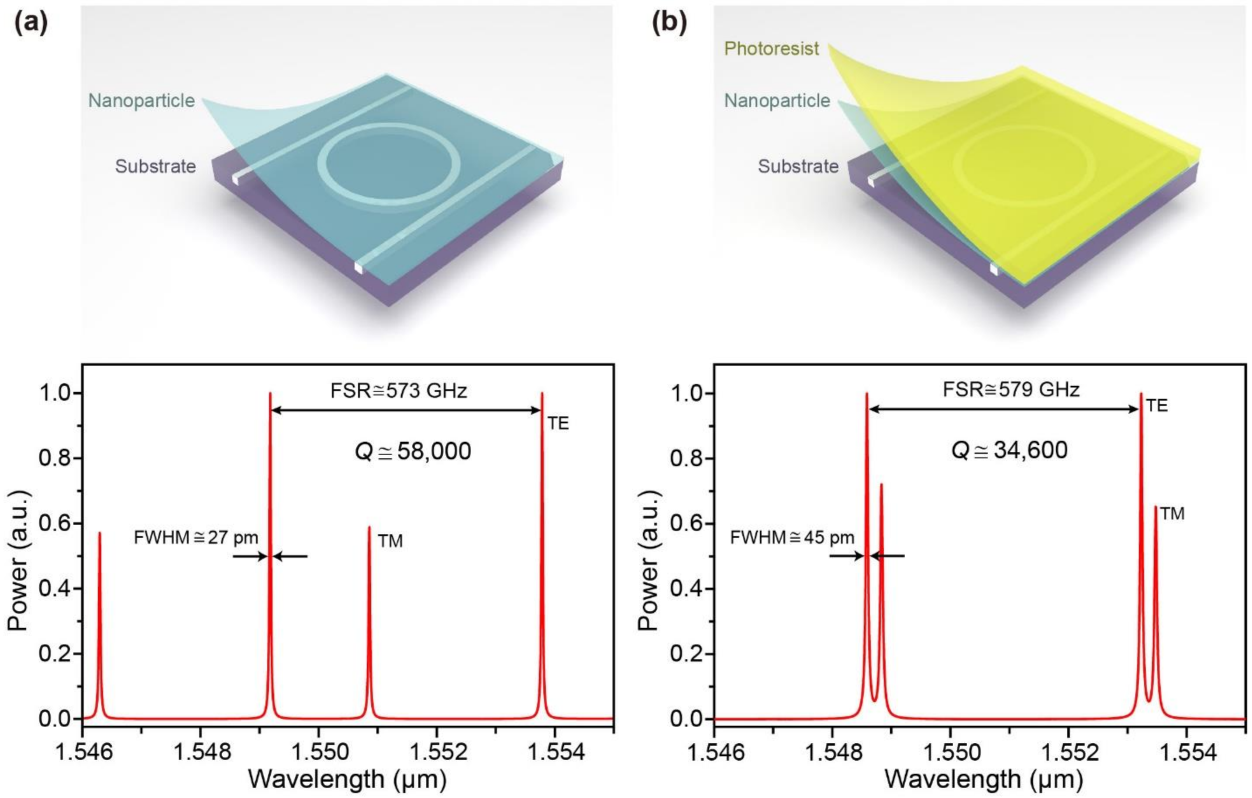

Supplementary Figure 18. Input–Drop port transfer function revealed the high wavelength selectively of microring resonator. Input–Drop port transfer function from λ = 1546 nm to 1555 nm for both transverse electric (TE) and magnetic (TM), showing Q-factors of ~58000 and ~34600, FSRs of 573 GHz and 579 GHz and FWHMs of 27 pm and 45 pm after deposition of the first nanoparticle layer (a) and the second SU-8 layer (b), respectively.

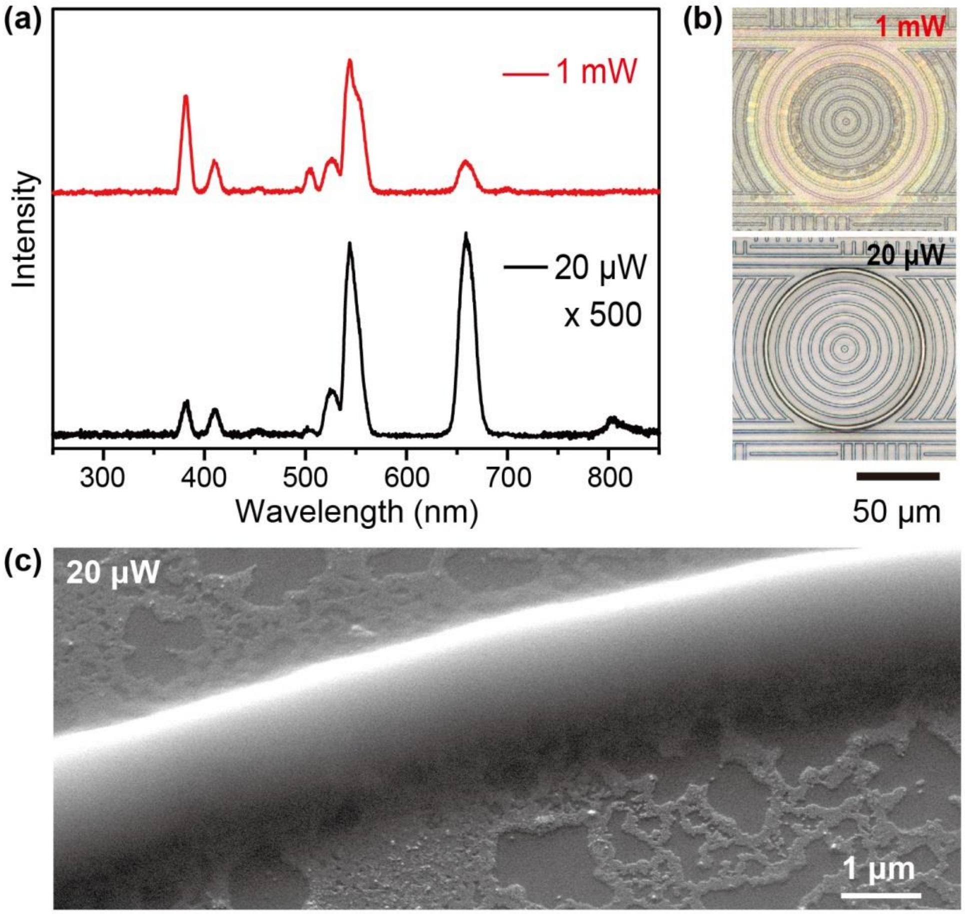

Supplementary Figure 19. Smoothness control of SU-8 layers developed on the microring resonator. (a) Emission spectra recorded under 20 µW and 1 mW excitation, showing the availability of UV emissions at reduced excitation powers. (b) The corresponding SU-8 patterns fabricated under 20 µW and 1 mW excitation, respectively. A relatively low level of excitation is needed for deposition of uniform SU-8 patterns. Excessive UV emission under high power excitation was found to cause uncontrollable photo-curing effect, leading to the formation of irregular SU-8 patterns. (c) 45-degree-tilted scanning electron micrograph shows smooth surface of the SU-8 pattern fabricated under 20 µW excitation. The irregular thin films surrounding the SU-8 pattern are ascribed to residual upconversion nanoparticles.

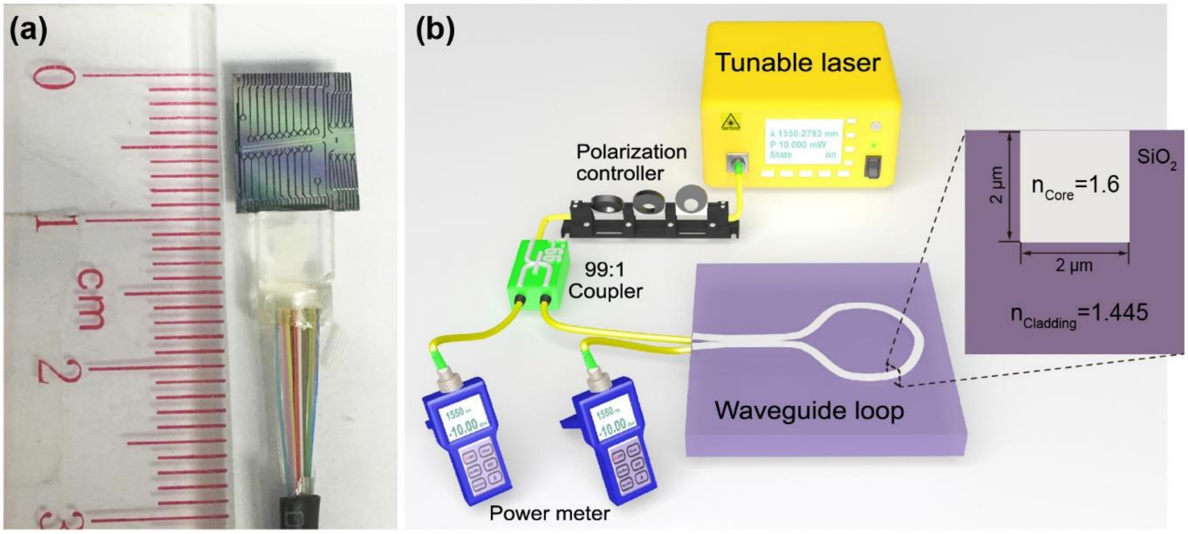

Supplementary Figure 20. Schematic diagram of the set-up for the waveguide loop platform. (a) The photograph of the device used in the experiment. (b) Geometry of the device and the setup for the experiment. The waveguide core is embedded in the SiO_2 substrate but having the top surface exposed to environment. In the experiment, the tunable laser was set to a fixed wavelength at 1550 nm. The polarization controller is tuned until maximum power was recorded at the power meter connected to the output of waveguide loop. The laser power was then set to the designed power level to cure the SU-8 pattern.

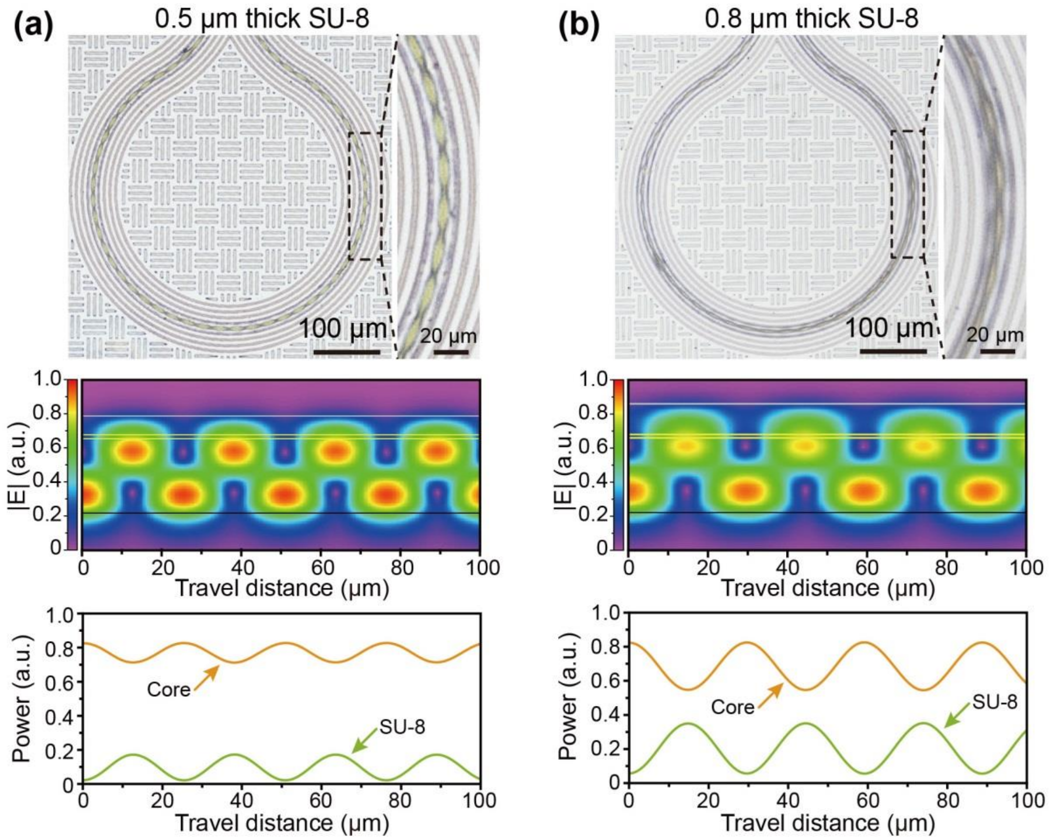

Supplementary Figure 21. Tuning period of SU-8 deposition through the control of coating thickness. Periodic patterns were fabricated with (a) 0.5 and (b) 0.8 μm thick SU-8 coatings. **Top:** Optical micrograph of the device after formation of SU-8 pattern. **Middle:** Distributions of the electric field amplitude along the propagation distance showing the beating between the odd and even modes. **Bottom:** Fractional power in the core region and in the SU-8 region of the waveguide as a function of propagation distance.

Supplementary References

[1] Wang, F., Deng, R. & Liu, X. Preparation of core−shell $NaGdF_4$ nanoparticles doped with luminescent lanthanide ions to be used as upconversion-based probes. *Nat. Protoc.* **9**, 1634−1644 (2014).

[2] Razzari, L. *et al*. CMOS-compatible integrated optical hyperparametric oscillator. *Nat. Photon.* **4**, 41−45 (2010).

[3] Ferrera, M. *et al*. Low-power continuous-wave nonlinear optics in doped silica glass integrated waveguide structures. *Nat. Photon.* **2**, 737−740 (2008).

[4] Kues, M. *et al*. Passively mode-locked laser with an ultra-narrow spectral width. *Nat. Photon.* **11**, 159−162 (2017).

[5] Kues, M. *et al*. On-chip generation of high-dimensional entangled quantum states and their coherent control. *Nature* **546**, 622−626 (2017).

[6] Pasquazi, A. *et al*. Sub-picosecond phase-sensitive optical pulse characterization on a chip. *Nat. Photon.* **5**, 618−623 (2011).

[7] Jin, L. *et al*. Mass-Manufactural Lanthanide-Based Ultraviolet B Microlasers. *Adv. Mater.* 1807079 (2018).

[8] Hutter, J. Excited state nuclear forces from the Tamm–Dancoff approximation to time-dependent density functional theory within the plane wave basis set framework. *J. Chem. Phys.* **118**, 3928−3934 (2003).

[9] Clark, S. J. *et al*. First principles methods using CASTEP. *Z. Krist.-Cryst. Mater.* **220**, 567−570 (2009).

[10] Hirata, S. & Head-Gordon, M. Time-dependent density functional theory within the Tamm–Dancoff approximation. *Chem. Phys. Lett.* **314**, 291−299 (1999).

[11] Lin, C. *et al*. Theoretical study on structure and sum-frequency generation (SFG) spectroscopy of styrene–graphene adsorption system. *J. Phys. Chem. C* **117**, 1754−1760 (2013).

[12] Lin, C. *et al*. Theoretical formulation and simulation of electronic sum-frequency generation spectroscopy. *J. Phys. Chem. C* **117**, 23797−23805 (2013).

[13] Kleinman, L. & Bylander, D. M. Efficacious form for model pseudopotentials. *Phys. Rev. Lett.* **48**, 1425−1428 (1982).

[14] Louie, S. G., Froyen, S. & Cohen, M. L. Nonlinear ionic pseudopotentials in spin-density-functional calculations. *Phys. Rev. B* **26**, 1738−1742 (1982).

[15] Grinberg, I., Ramer, N. J. & Rappe, A. M. Transferable relativistic Dirac-Slater pseudopotentials. *Phys. Rev. B* **62**, 2311−2314 (2000).

[16] Rappe, A. M., Rabe, K. M., Kaxiras, E. & Joannopoulos, J. D. Optimized pseudopotentials. *Phys. Rev. B* **41**, 1227−1230 (1990).

[17] Huang, B. L. 4f fine-structure levels as the dominant error in the electronic structures of binary lanthanide oxides. *J. Comput. Chem.* **37**, 825−835 (2016).

[18] Huang, B. L. Intrinsic deep hole trap levels in Cu_2O with self-consistent repulsive Coulomb energy. *Solid State Commun.* **230**, 49−53 (2016).

[19] Huang, B. L. Native point defects in CaS: focus on intrinsic defects and rare earth ion dopant levels for up-converted persistent luminescence. *Inorg. Chem.* **54**, 11423−11440 (2015).

[20] Huang, B. L. Strong compensation hinders the p-type doping of ZnO: a glance over surface defect levels. *Solid State Commun.* **237-238**, 34−37 (2016).

[21] Huang, B. L. Unraveling energy conversion modeling in the intrinsic persistent upconverted luminescence of solids: a study of native point defects in antiferromagnetic Er_2O_3. *Phys. Chem. Chem. Phys.* **18**, 13564−13582 (2016).

[22] Huang, B. L. *et al*. Fundamental view of electronic structures of β-$NaYF_4$, β-$NaGdF_4$, and β-$NaLuF_4$. *J. Phys. Chem. C* **120**, 18858−18870 (2016).

[23] Huang, B. L. The screened pseudo-charge repulsive potential in perturbed orbitals for band calculations by DFT+U. *Phys. Chem. Chem. Phys.* **19**, 8008−8025 (2017).

[24] Huang, B. L., Gillen, R. & Robertson, J. Study of CeO_2 and its native defects by density functional theory with repulsive potential. *J. Phys. Chem. C* **118**, 24248−24256 (2014).

[25] Huang, B. L. Superiority of DFT+U with non-linear core correction for open-shell binary rare-earth metal oxides: a case study of native point defects in cerium oxides. *Philos. Mag.* **94**, 3052−3071 (2014).

[26] Huang, B. L. & Sun, M. Energy conversion modeling of the intrinsic persistent luminescence of solids via energy transfer paths between transition levels. *Phys. Chem. Chem. Phys.* **19**, 9457−9469 (2017).

[27] Huang, B. L. Doping of RE ions in the 2D ZnO layered system to achieve low-dimensional upconverted persistent luminescence based on asymmetric doping in ZnO systems. *Phys. Chem. Chem. Phys.* **19**, 12683−12711 (2017).

[28] Chen, Q. *et al*. Confining excitation energy in Er^{3+}-sensitized upconversion nanocrystals through Tm^{3+}-mediated transient energy trapping. *Angew. Chem. Int. Ed.* **56**, 7605−7609 (2017).

[29] Huang, B. L. *et al*. Unravelling the energy transfer of Er^{3+}-self-sensitized upconversion in Er^{3+}-Yb^{3+}-Er^{3+} clustered core@shell nanoparticles. *Nanoscale* **9**, 18490−18497 (2017).
